# The combination of a glucagon-like peptide-1 and amylin receptor agonists reduces alcohol consumption in both male and female rats

**DOI:** 10.1017/neu.2024.58

**Published:** 2024-12-06

**Authors:** Cajsa Aranäs, Christian E. Edvardsson, Lindsay Zentveld, Daniel Vallöf, Sarah Witley, Maximilian Tufvesson-Alm, Olesya T. Shevchouk, Jesper Vestlund, Elisabet Jerlhag

**Affiliations:** Department of Pharmacology, Institute of Neuroscience and Physiology, The Sahlgrenska Academy at the University of Gothenburg, Gothenburg, Sweden

**Keywords:** Gut-brain axis, addiction, appetite-regulation, reward, dopamine

## Abstract

**Objective:**

Combining different pharmaceuticals may be beneficial when treating disorders with complex neurobiology, including alcohol use disorder (AUD). The gut-brain peptides amylin and GLP-1 may be of potential interest as they individually reduce alcohol intake in rodents. While the combination of amylin receptor (AMYR) and glucagon-like peptide-1 receptor (GLP-1R) agonists have been found to decrease feeding and body weight in obese male rats synergistically, their combined impact on alcohol intake is unknown.

**Methods:**

Therefore, the effect of the combination of an AMYR (salmon calcitonin (sCT)) and a GLP-1R (dulaglutide) agonist on alcohol intake in rats of both sexes was explored in two separate alcohol-drinking experiments. The first alcohol-drinking experiment evaluated the potential of adding sCT to an ongoing dulaglutide treatment, whereas the second alcohol-drinking experiment examined the effect when adding sCT and dulaglutide simultaneously.

**Results:**

When adding sCT to an ongoing dulaglutide treatment, a reduction in alcohol intake was observed in both male and female rats. However, when combining sCT and dulaglutide simultaneously, an initial reduction in alcohol intake was observed in rats of both sexes, whereas tolerance towards treatment was observed. In both alcohol-drinking experiments, this treatment combination consistently decreased food consumption and body weight in males and females. While the treatment combination did not affect inflammatory mediators, the gene expression of AMYR or GLP-1R, it changed fat tissue morphology.

**Conclusions:**

Further investigation needs to be done on the combination of AMYR and GLP-1R agonists to assess their combined effects on alcohol intake.

Significant outcomes

- Adding sCT to an ongoing dulaglutide treatment reduces alcohol intake in male and female rats
- Tolerance towards the ability of the combination to reduce alcohol intake is developed when adding the drugs simultaneously
- The combination of sCT and dulaglutide reduces food intake and body weight in rats of both sexes

Limitations

- Underlying mechanisms were studied, but not identified
- The design of the two alcohol-drinking experiments diverged
- sCT, a dual amylin and calcitonin receptor agonist, rather than a selective amylin receptor agonist was used

## Introduction

Alcohol use disorder (AUD) is a prevalent neuropsychiatric disorder associated with an enhanced risk of morbidity and mortality (Nestler, 2001, Carvalho *et al*., 2019). Besides the individual harm, it contributes to a substantial burden on society and has profound socio-economic consequences (Ferrari *et al*., 2014). The chronic nature of AUD is characterised, among other factors, by escalated alcohol intake, and recurring cycles of relapse (Koob, 2014). AUD has a multifaceted aetiology (for review see (Berke and Hyman, 2000; Nestler, 2001)), underscoring the challenges faced in achieving effective pharmacological interventions. While various neurobiological mechanisms have been implied in the pathophysiology, gut-brain peptides glucagon-like peptide-1 (GLP-1) and amylin have gained recent attention (for review see (Tufvesson-Alm *et al*., 2022)). Specifically, alcohol intake is lower in both male and female rats after administration of GLP-1R agonists (Egecioglu *et al*., 2013, Shirazi *et al*., 2013, Sørensen *et al*., 2016, Vallöf *et al*., 2016, Marty *et al*., 2020, Vallöf *et al*., 2020, Aranäs *et al*., 2023). Dulaglutide, a GLP-1R agonist, has shown promising effects in reducing alcohol intake in rats of both sexes (Vallöf *et al*., 2020). On a similar note, the amylin receptor (AMYR) and calcitonin receptor (CTR) agonist, salmon calcitonin (sCT), decreases alcohol intake and prevents alcohol deprivation effect in male rats (Kalafateli, Vallöf *et al*., 2019, Kalafateli, Vallöf *et al*., 2019). Together these studies show that GLP-1R and AMYR agonists both regulate feeding and alcohol consumption, highlighting similarities in the modulation of these behaviours.

To date, four pharmacological interventions, nalmefene, naltrexone, acamprosate and disulfiram, have been approved for the treatment of AUD. However, there is a need for new treatment options as the currently approved AUD medications display limited efficacy (for review see (Heilig and Egli, 2006; Koob, 2010)). In the pursuit of novel therapeutics, the combination of agents that separately reduce alcohol intake has been suggested to enhance efficacy. As such, the combination of naltrexone and/or antismoking agents (varenicline, bupropion) synergistically reduces alcohol intake in rodents (Nicholson *et al*., 2018, Zhou *et al*., 2019, Söderpalm *et al*., 2020, Ray *et al*., 2021). The strategy to combine different medications has also gained attention when it comes to other heterogeneous disorders, including obesity. Indeed, combining gut-brain peptides synergistically reduces body weight and food intake. This is evident as liraglutide (a GLP-1R agonist) in combination with sCT synergistic-like reduces energy intake and body weight in rats, as well as Exendin-4 (another GLP-1R agonist) together with sCT synergistically lowers food intake in non-human primates. Furthermore, a case study indicates that combining semaglutide (a GLP-1R agonist) or dulaglutide with pramlinitide (another AMYR agonist) decreases body weight, an effect that potentially could be synergistic (Bello *et al*., 2010, Camilleri and Acosta, 2018, Liberini *et al*., 2019, Wong *et al*., 2023). These studies further revealed that the treatment outcome in these studies were influenced by how these drugs were combined (Bello *et al*., 2010, Camilleri and Acosta, 2018, Liberini *et al*., 2019, Wong *et al*., 2023). Given that the combination of GLP-1R and AMYR agonists reduces feeding, and as these gut-brain peptides individually reduce alcohol intake we hypothesise that GLP-1R and AMYR agonists, when used together, tentatively could synergistic-like reduce alcohol intake in male and female rats. This study therefore evaluated the combination of dulaglutide, which individually has demonstrated significant alcohol-reducing effects in both sexes, with sCT, as it has shown a promising impact on alcohol-related behaviours in rodents (Kalafateli, Vallöf *et al*., 2019, Kalafateli, Vallöf *et al*., 2019, Vallöf *et al*., 2020). The first alcohol-drinking experiment evaluated the potential of adding sCT to an ongoing dulaglutide treatment, whereas the second alcohol-drinking experiment examined th

e effect when adding sCT and dulaglutide simultaneously. We further examined the impact of this treatment combination on food intake and body weight and used molecular experiments in attempts to define underlying mechanisms.

## Material and methods

### Animals

Post-pubertal male (approximately 250 g at arrival) and female (approximately 190 g at arrival) Rcc/Han Wistar rats (Envigo, Horst, the Netherlands) were used as strain displays high and stable alcohol intake (Simms *et al*., 2008, Vallöf *et al*., 2020, Kalafateli *et al*., 2021) causing pharmacologically relevant blood alcohol concentrations (Simms *et al*., 2008). Upon arrival, all rats habituated to the animal facilities for one week and were thereafter housed individually. The rats were maintained at a reversed light/dark cycle (lights out at 10 am). The studies were approved by the Ethics Committee for Animal Experiments, Gothenburg, Sweden (1556/18, 1457/18, and 3276/20), and were carried out by ARRIVE guidelines. All rats were euthanized using an overdose of Isoflurane followed by decapitation. 6-10 rats per treatment were used as this group size provides enough power to detect an effect if it is present.

### Drugs

An alcohol solution of 20% (95% Ethanol; Solveco, Stockholm, Sweden; diluted in tap water) was used. The long-acting GLP-1R agonist, dulaglutide (Trulicity®; Kronans Apotek, Gothenburg, Sweden), was diluted in vehicle (0.9% NaCl) and thereafter administrated subcutaneously. Given that dulaglutide is long-acting, with a half-life of 2 days it was administered once weekly (Glaesner *et al*., 2010). The AMYR and CTR agonist, sCT (Tocris Bioscience; Bristol, United Kingdom), was diluted in vehicle (0.9% NaCl), and was injected intraperitoneally daily. As in previous studies, dulaglutide (0.1 mg/kg) and sCT (5 µg/kg) were injected 60 minutes before alcohol exposure as they have been found to reduce alcohol intake in rats when administered individually (Kalafateli, Vallöf *et al*., 2019, Vallöf *et al*., 2020).

### Intermittent access paradigm

Two separate alcohol-drinking experiments were conducted in male and female rats. According to established protocol, the rats had access to one alcohol and one water bottle for three 24-hour sessions (*i.e.* alcohol-drinking session) per week (Monday, Wednesday, and Friday) and two water bottles the days in between (Vallöf *et al*., 2016, Vallöf *et al*., 2020). The alcohol intake was measured before initiation of drug injections (baseline drinking), allowing the division of rats into treatment groups with similar alcohol intake during the baseline period (data not shown). The rats’ body weights were similar prior to the start of treatment (data not shown). Throughout the experiments, the alcohol intake, food intake, body weight (weight after treatment - weight before treatment), alcohol preference (the ratio of alcohol over the total fluid intake), water intake and total fluid intake (alcohol and water intake together) were measured.

### Alcohol-drinking experiment 1

It has been shown that the most beneficial weight reduction occurs when adding an AMYR agonist to a prolonged GLP-1R agonist treatment (Liberini*et al*., 2019). The first alcohol-drinking experiment therefore evaluated the potential of adding sCT to an ongoing dulaglutide treatment. Thus, male (*n*= 40) and female (*n*= 40) rats consumed alcohol for one day (baseline drinking) before treatment initiation. During the next ten weeks, dulaglutide or vehicle was injected weekly. Then sCT was co-administered with dulaglutide for an additional two weeks (*i.e.* sessions 1-6). For ethical reasons, the rats cannot be in the experiment more than 14 weeks, necessitating that treatment begins before an extended baseline period of alcohol. The rats were euthanized after two 24-hour water-drinking sessions, and *ex vivo* tissues (brain, fat tissues, trunk blood converted to serum) were collected and stored at −80°C until further processing (see ‘*ex vivo* measurements’ for detailed information).

### Alcohol-drinking experiment 2

The second alcohol-drinking experiment examined the impact of the simultaneous administration of sCT and dulaglutide on alcohol intake in rats of both sexes. After consumption of alcohol for ten weeks (baseline drinking), male (*n*= 24) and female (*n*= 24) were deprived of alcohol for 13 days. Before alcohol was reintroduced, the rats were treated with dulaglutide weekly and sCT daily for two additional weeks. This experiment evaluated treatment impact on relapse drinking (*i.e.* delta alcohol intake) at the two first sessions and alcohol consumption during the remaining sessions (sessions 3-7). The rats were euthanized after one 24-hour water-drinking session, and their length (without tail), as well as body weight, were measured. The different post-mortem analyses conducted in experiments 1 and 2 are not intended for direct comparison, effects are long-lasting for dulaglutide, and the difference in time for sacrifice should therefore not affect the outcome. *Ex vivo* tissues (fat tissues, spleen, pancreas, liver, and calf muscle) were collected and weighed. Blood glucose measurements were taken from trunk blood (collected from the neck after decapitation) at the time of sacrifice. Gonadal and subcutaneous fat tissues were stored in histofix (Histolabs, Askim, Sweden) until further processing (see ‘*ex vivo* measurements’ for detailed information).

### Ex vivo measurements

#### Alcohol-drinking experiment 1 - Effects of the combination treatment on gene expression in brain and fat tissues and the serum levels of proteins and peptides

After the rats in experiment 1 were euthanized, the brains were removed and placed in a brain-slicing matrix on dry ice. Thereafter, the nucleus accumbens shell (NAcS), ventral tegmental area (VTA), laterdorsal tegmental nucleus (LDTg), and paraventricular thalamus (PVT) were rapidly punched out and stored at −80°C. In addition to the brains, fat tissues were collected. Renal (defined as the complete peritoneal and retroperitoneal fat pads combined), subcutaneous (a predetermined groin section), gonadal- and brown adipose fat tissue were obtained by dissection immediately following the sacrifice of the rat. Although subcutaneous fat is distributed throughout the body, it was only dissected from one predefined area due to technical limitations to full dissection. The dissection of each distinct fat tissue was consistently carried out by the same individual to ensure uniformity across all rats. After dissection, all fat tissues were weighed and stored at −80°C.

To enable gene expression analysis, the extraction, quality check, and determination of RNA concentration as well as reverse transcription into cDNA were conducted as previously described (Kalafateli, Vallöf *et al*., 2019). The quantitative real-time PCR (qRT-PCR) was conducted at TATAA Biocenter AB (Gothenburg, Sweden) at an IntelliQube^TM^ (Douglas Scientific, Alexandra, MN, USA) (Kalafateli, Vallöf *et al*., 2019). Corrected CT values were obtained by addressing gDNA contamination using ValidPrime^TM^ (TATAA Biocenter AB) technology. The impact of the combination treatment on the expression of AMYR and GLP-1-related genes in brain areas central for reward processing (for review see, (Söderpalm and Ericson, 2013; Engel and Jerlhag, 2014, Millan *et al*., 2017)) was examined. The gene expression of *CTR,* the main component of the AMYR (Rn00587525_m1), and the receptor activity-modifying protein 1 (*RAMP1*; Rn01427056_1). was measured. *RAMP1* was selected as its expression in NAcS has been associated with high alcohol intake in male rats (Kalafateli, Vallöf *et al*., 2019), and therefore, the expression of RAMP2 and RAMP3 was not evaluated. Additionally, the expression of *GLP-1R* (Rn00562406_m1), was evaluated as it previously has been associated with high alcohol intake in male rats (Vallöf, Kalafateli *et al*., 2019). Besides reducing alcohol and food intake, the combination treatment reduced the body weight in rats. The possibility that the combination treatment alters the expression of genes central to fat metabolism (Liang and Ward, 2006, Seale *et al*., 2008, Harms *et al*., 2014, Bargut *et al*., 2017, Richard *et al*., 2017, Ikeda and Yamada, 2020) was therefore evaluated in fat tissues from these rats. The gene expression of *PGc1α* (RN01427056_m1), *PRDM16* (RN00587525_m1), *UCP1* (RN00562406_m1), *PPARγ* (RN00440945_m1) was investigated in brown adipose, subcutaneous, and gonadal fat tissue. In both gene expression experiments, *GAPDH* (RN01775763_g1) was selected as the reference gene and the gene expression values were analysed with relative quantification (Livak and Schmittgen, 2001).

Following decapitation, trunk blood from the rat’s neck was collected in serum tubes (Z-gel tubes with clotting activator; Sarstedt, N FC;mbrecht, Germany). Aliquots of serum were stored at −80°C and transferred into duplicates in multi-well plates as described before (Vestlund and Jerlhag, 2020).

Previous studies have shown that GLP-1R effects on cocaine-related behaviours was associated with the down-regulation of inflammatory cytokines (Zhu *et al*., 2021). Moreover, amylin alters the levels of pro-inflammatory cytokines, which suggests that amylin could trigger an inflammatory response (Srodulski *et al*., 2014, Gillies *et al*., 2017). We therefore hypothesise that the combination treatment may influence cytokine levels in serum. However, without a pair-fed control group can we only investigate the descriptive changes in the gene expression and not conclude whether alcohol intake or weight influences gene expression or if gene expression influences alcohol intake or weight. A panel of cytokines was therefore measured in serum from the alcohol-drinking rats from experiment 1. We further hypothesise that the combination treatment alters the serum of GLP-1, amylin, and corticosterone since gut-brain peptides have well-documented effects on these hormones (Terrill *et al*., 2018, Vestlund and Jerlhag, 2020).

ELISA kits were used to measure the serum levels of corticosterone (ADI-900-097, AH diagnostics, Stockholm, Sweden), GLP-1 (RAB0201, Sigma-Aldrich, Saint-Louise, Missouri, USA), and amylin (NBP2-76735, Novus Biologicals, Abingdon, UK). The optical density of the samples was detected by a microplate photometer (Multiskan GO, Thermo Fisher Scientific, Darmstadt, Germany) at a defined wavelength (405 nm; Corticosterone, 450 nm; GLP-1 and amylin). The fluorescence Bio-Plex Pro^TM^ rat Cytokine Assay kit (10014905, Bio-Rad, Hercules, CA, USA) was used to quantify the cytokines levels. The fluorescence intensity of these samples was detected in a Bio-Plex 200 system (Bio-Rad) to determine the concentration of multiple cytokines in one well.

#### Alcohol-drinking experiment 2 - Effects of the combination treatment on the weight, as well as cellular and tissue structure of fat tissues

After the termination of alcohol-drinking experiment 2, the retroperitoneal, peritoneal, subcutaneous (a predetermined groin section), gonadal- and brown adipose fat tissues were collected by dissection and subsequently weighed. The dissection of each specific fat tissue was consistently performed by the same individual to maintain consistency across all rats. As the combination treatment reduces the body weight and the weight of gonadal or subcutaneous fat tissues in rats, we hypothesise that treatment affects the cellular and tissue structure of these fat tissues. Therefore, gonadal and subcutaneous fat tissues were stored in histofix (Histolabs, Askim, Sweden), and sliced at Histocenter AB (Gothenburg, Sweden). Cellular and tissue structures were visualised by haematoxylin-eosin stained with Mayer’s core staining at Histocenter AB. Thereafter, the number and area of cells, and connective tissue (the contrast between cells) were measured with Fiji Image J (Fiji, Wisconsin, USA) from three randomly selected slices per tissue and rat.

To identify profound inflammation effects by treatments in experiment 2, the spleen and pancreas were collected and weighed. To exclude the possibility that the treatment reduces blood glucose levels, which is known for GLP-1R and AMYR agonists in diabetes studies (for review see, (Scherbaum, 1998; Meier, 2012)) the blood glucose levels (CONTOUR® XT; Ascensia Diabetes Care Sweden AB, Solna, Sweden) were measured in trunk blood (collected from the neck after decapitation). As the combination treatment reduces the rat’s body weight additional measurements were made to elucidate tentative mechanisms behind this reduction. The rat’s length (without tail) was measured to exclude the possibility that impaired growth leads to a reduction in body weight. Additionally, the liver and calf muscles were collected by dissection and weighed to explain the observed weight loss.

### Statistical analysis

A repeated two-way ANOVA analysed the data from repeated alcohol-drinking experiments. The data from *ex vivo* experiments were analysed with a one-way ANOVA test or unpaired *t*-test, followed by either Tukey’s or Bonferroni’s post-hoc test. The probability of *P* < 0.05 is considered statistically significant and adjusted for multiple testing using statistical hypothesis testing (Tukey’s or Bonferroni’s). All statistical analysis was performed using Prism 10.0 (GraphPad Software, Inc., CA, USA). In the present drinking experiments, the pre-set exclusion criteria were abnormal rat behaviour, leaking bottles, and weight loss >15%, but no rats were excluded.

## Results

### Effects on alcohol intake in male rats from experiments 1 and 2

**Figure 1.**
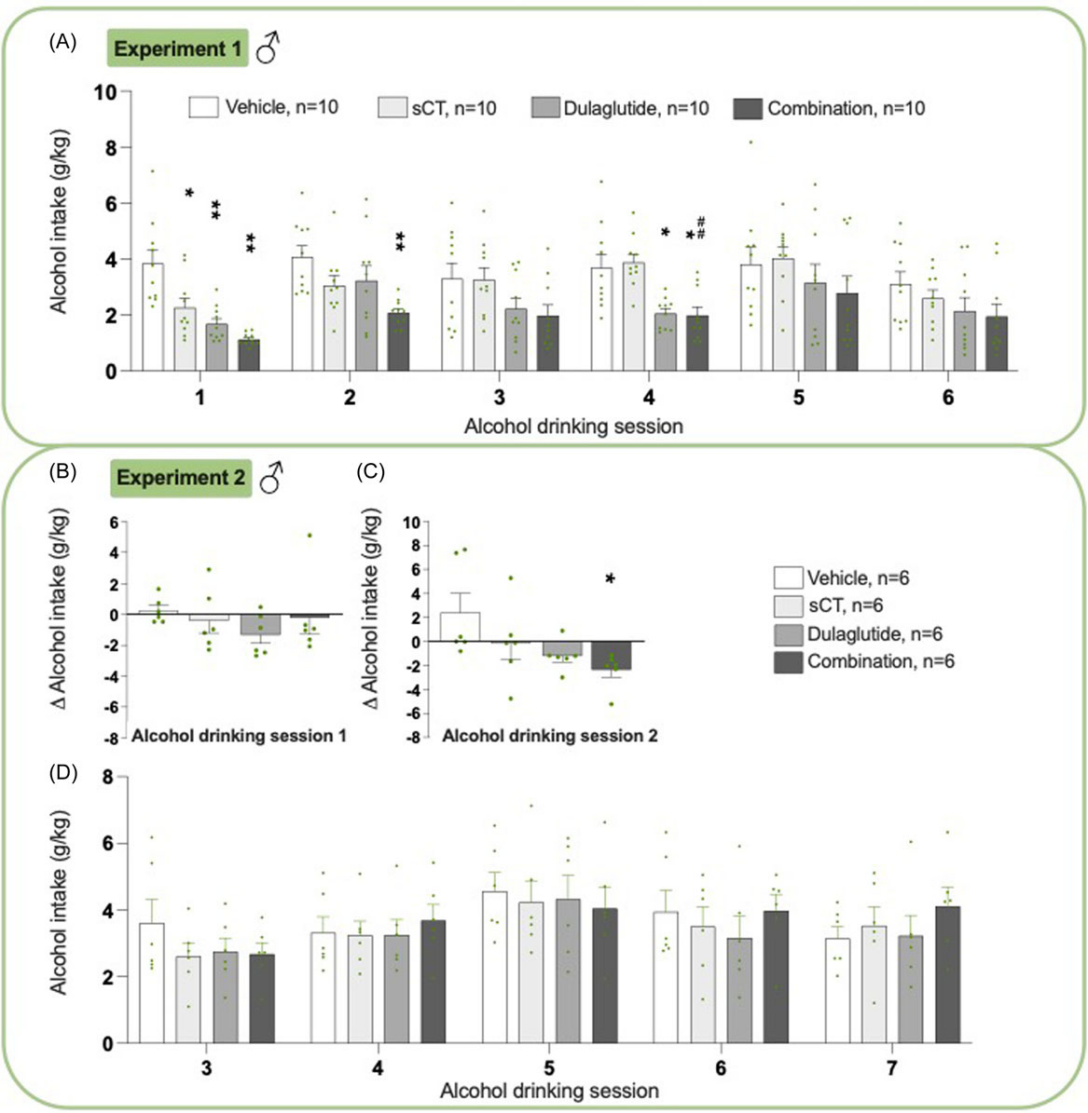
**Treatment effect on alcohol intake in male rats**. (A) During experiment 1, the combination treatment decreased alcohol intake compared to both vehicle (evident at alcohol drinking sessions 1, 2, and 4) and salmon calcitonin (sCT) (evident at alcohol drinking session 4). Additionally, when administered as monotherapy, both dulaglutide (alcohol drinking session 1 and 4) and sCT (alcohol drinking session 1) exhibited a reduction in alcohol intake compared to vehicle. However, this decline in alcohol consumption was found on fewer days compared to the combination treatment. (B) In experiment 2, the combination of dulaglutide and sCT did not alter the delta alcohol intake at alcohol drinking session 1, (C) whereas it decreased the delta alcohol intake at alcohol drinking session 2. (D) However, the combination treatment did not alter the alcohol intake during alcohol drinking sessions 3-7. Moreover, in experiment 2, neither dulaglutide nor sCT altered the alcohol intake. Data are presented as mean ± standard error of the mean (SEM), significant data are illustrated by *P < 0.05 **P < 0.01, compared to vehicle, ##P < 0.01 compared to sCT.

Before sCT was added to the ongoing dulaglutide treatment, the alcohol intake was lowered by dulaglutide (Supplementary Figure 1A, G) and impacted other measured parameters (Supplementary Figure 1B-G). When adding sCT to the ongoing dulaglutide treatment, there was an overall reduction in alcohol intake (*P*= 0.0096, two-way ANOVA, Fig. 1A, Supplementary Table 1). Specifically, the combined treatment reduced alcohol consumption in sessions 1 (*P*< 0.01), 2 (*P*< 0.01), and 4 (*P*< 0.05) compared to vehicle. Additionally, the combined treatment had a greater reduction in alcohol intake compared to sCT at session 4 (*P*< 0.01). On the contrary, monotherapy with sCT decreased alcohol intake at session 1 (*P*< 0.05), and dulaglutide reduced alcohol intake at session 1, *P* < 0.01 and 4, *P* < 0.05.

When sCT and dulaglutide were injected simultaneously, there was no overall effect on the alcohol intake at alcohol-drinking session 1 (*P*= 0.5175, one-way ANOVA, Fig. 1B, Supplementary Table 1). However, there was an overall reduction in the delta alcohol intake at alcohol-drinking session 2 (*P* = 0.0406, one-way ANOVA, Fig. 1C). Specifically, compared to vehicle the combination decreased the delta alcohol intake (*P* = 0.0315), whereas neither sCT nor dulaglutide influenced the alcohol intake. Moreover, there was no overall effect of treatment on alcohol intake at alcohol-drinking sessions 3–7 (Fig. 1D, *P*= 0.9195 two-way ANOVA).

### Effects on alcohol intake in female rats from experiments 1 and 2

**Figure 2.**
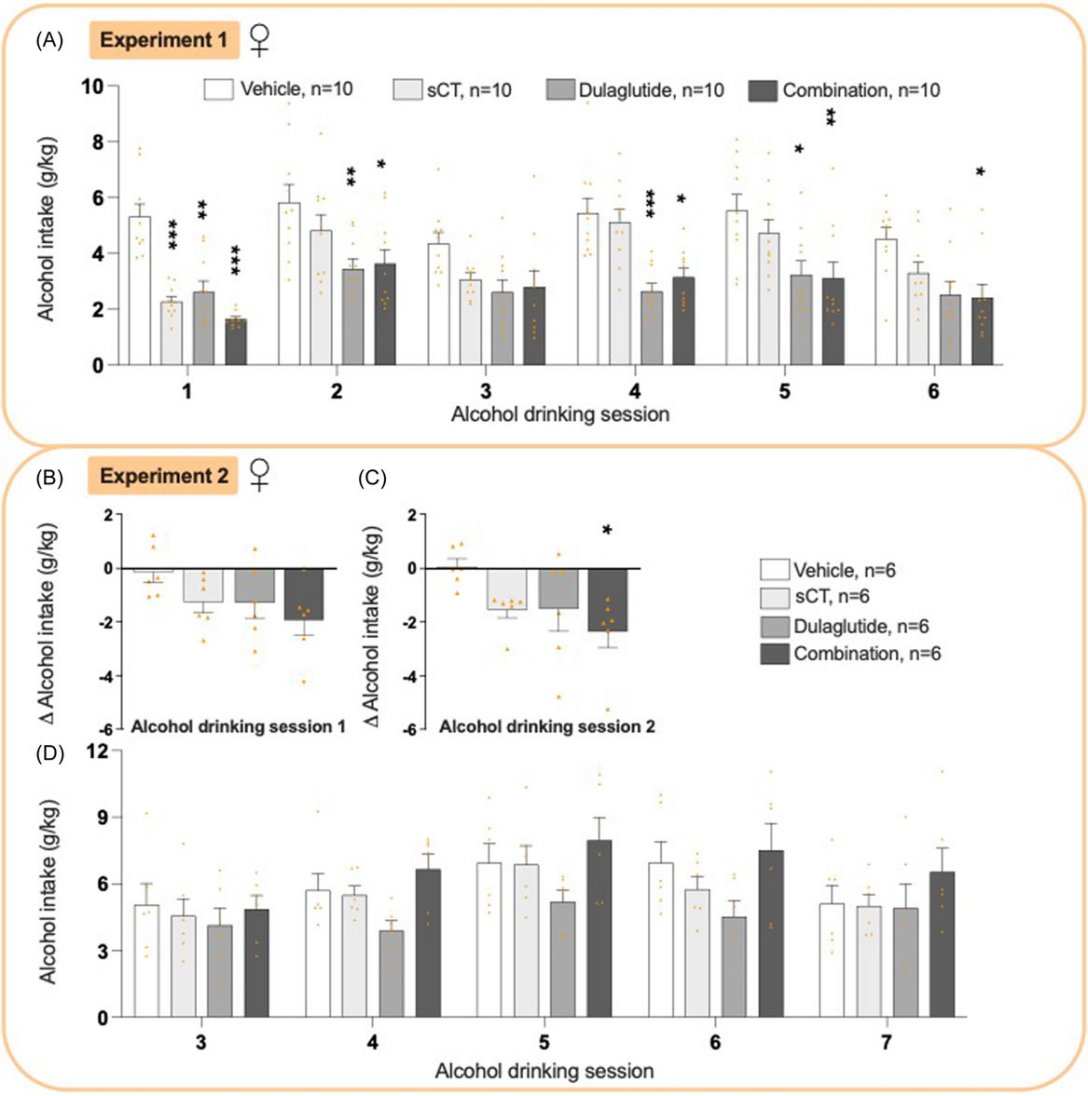
**Treatment effect on alcohol intake in female rats**. (A) In experiment 1, the combined treatment decreased alcohol intake compared to vehicle (at sessions 1, 2, 4, 5). When administered individually, dulaglutide (sessions 1, 2, 4) and salmon calcitonin (session 1) decreased the alcohol intake compared to vehicle. However, the combination reduced drinking at more alcohol drinking sessions than the monotherapies. (B) In experiment 2, neither treatment reduced the delta alcohol intake at session 1. (C) The combination, but neither monotherapy decreased the delta alcohol intake at session 2. (D) Moreover, neither treatment altered the alcohol intake during sessions 3-7. Data are presented as mean ± SEM, significant data are illustrated by *P < 0.05, **P < 0.01, ***<0.0001 compared to vehicle.

Before adding sCT to dulaglutide treatment, dulaglutide alone lowered alcohol intake (Supplementary Figure 2A, G) and impacted other measured parameters (Supplementary Figure 2B-G). When adding sCT to the ongoing dulaglutide treatment, there was an overall reduction in alcohol intake in female rats (*P*= 0.0002, two-way ANOVA, Fig. 2A, Supplementary Table 1), with significant decreases during session 1 *P* < 0.001, 2 and 4 *P* < 0.05, 5 *P* < 0.01, 6 *P* < 0.05. In contrast, monotherapy with sCT decreased alcohol intake at session 1 and dulaglutide decreased alcohol intake at sessions 1–2 *P* < 0.01, 4 *P* < 0.001, and 5 *P* < 0.05.

Simultaneous sCT and dulaglutide treatment had no overall effect on alcohol intake at session 1 (*P*= 0.1095, one-way ANOVA, Fig. 2B, Supplementary Table 1), but overall reduced alcohol intake in session 2 (*P*= 0.0387, one-way ANOVA, Fig. 2C). Specifically, compared to vehicle the combination treatment lowered the alcohol intake (*P*= 0.0263), with no effects seen in sessions 3–7 (Fig. 2D, *P*= 0.1460 two-way ANOVA).

### Effects on food intake in male rats from experiments 1 and 2

**Figure 3.**
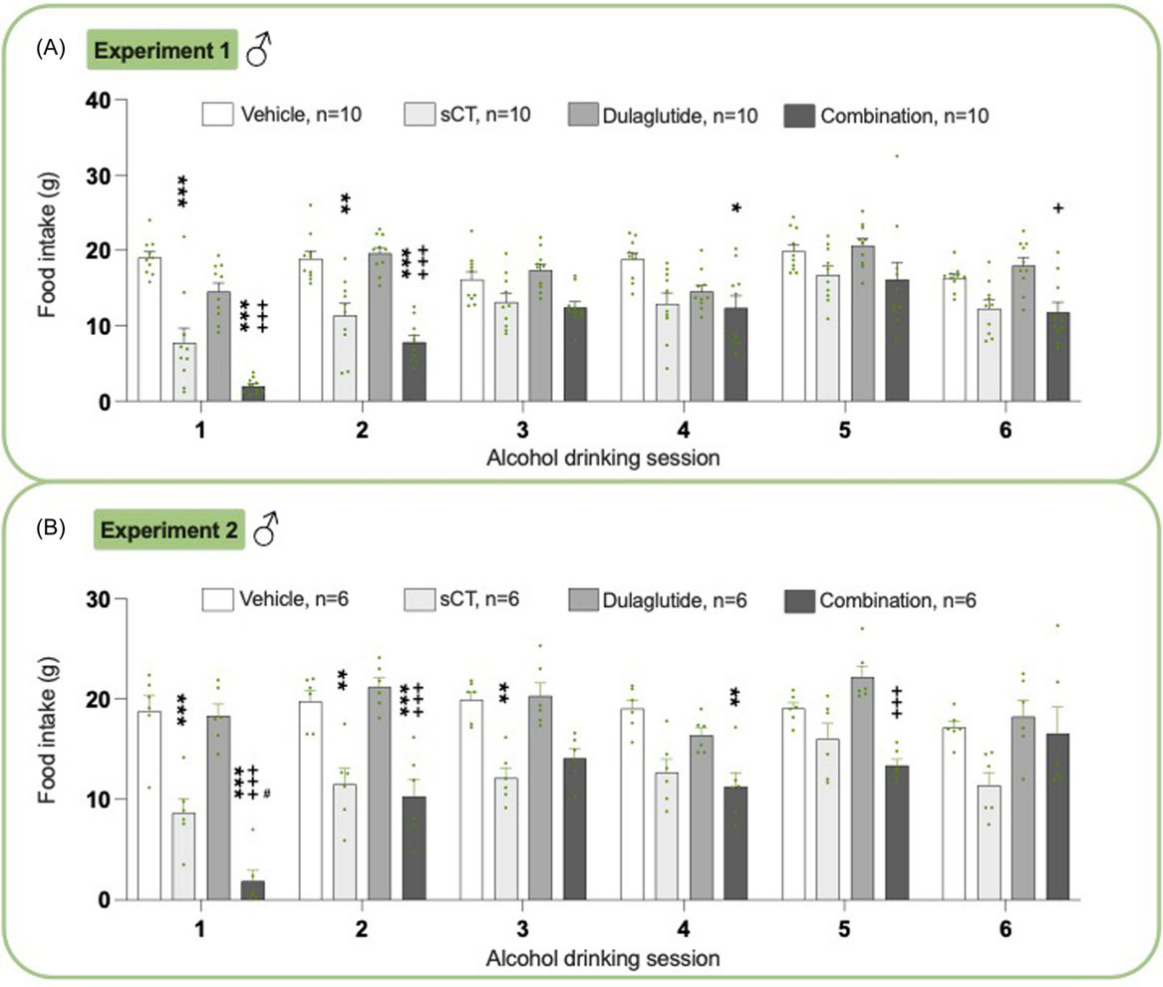
**Treatment effect on food intake in male rats**. (A) Within experiment 1, the combination treatment decreased food intake compared to either vehicle (at sessions 1, 2, 4) or dulaglutide (at sessions 1, 2, 6). When administered individually, salmon calcitonin (sCT) reduced food intake compared to vehicle at sessions 1 and 2. (B) In experiment 2, the combination treatment declined food intake compared to vehicle (alcohol drinking session 1, 2, 4), sCT (alcohol drinking session 1), and dulaglutide (session 1, 2, 5). Compared to vehicle, sCT did decrease the food intake at sessions 1, 2, and 3. Both in experiment 1 and 2, the combination treatment reduced food intake at more sessions than the monotherapies. Data are presented as mean ± SEM, significant data are illustrated by *P < 0.05, **P < 0.01, ***<0.0001 compared to vehicle, +P < 0.05, +++P < 0.0001 compared to dulaglutide, #P < 0.05 compared to sCT.

In male rats of Experiment 1, treatment caused an overall reduction in food intake (*P*< 0.0001 two-way ANOVA, Fig. 3A, Supplementary Table 2). Specifically, the combination treatment reduced food intake at sessions 1–2 (*P*< 0.001) and 4 (*P*< 0.05) compared to vehicle as well as to dulaglutide at sessions 1–2 (*P*< 0.001) and 6 (*P*< 0.05). Dulaglutide did not affect food intake, while sCT decreased it at sessions 1 (*P*< 0.001) and 2 (*P*< 0.01).

Similarly, in experiment 2, food intake was overall lowered by treatment (*P*< 0.0001 two-way ANOVA, Fig. 3B, Supplementary Table 2). Specifically, the combination reduced the food intake compared to vehicle at sessions 1–2 (*P* < 0.001) and 4 (*P*< 0.01), sCT at session 1 (*P*< 0.05) as well as dulaglutide at sessions 1–2 and 5 (*P*< 0.001). While dulaglutide had no impact on feeding, sCT reduced food intake at sessions 1 (*P*< 0.001) and 2 (*P*< 0.01).

### Effects on food intake in female rats from experiments 1 and 2

**Figure 4.**
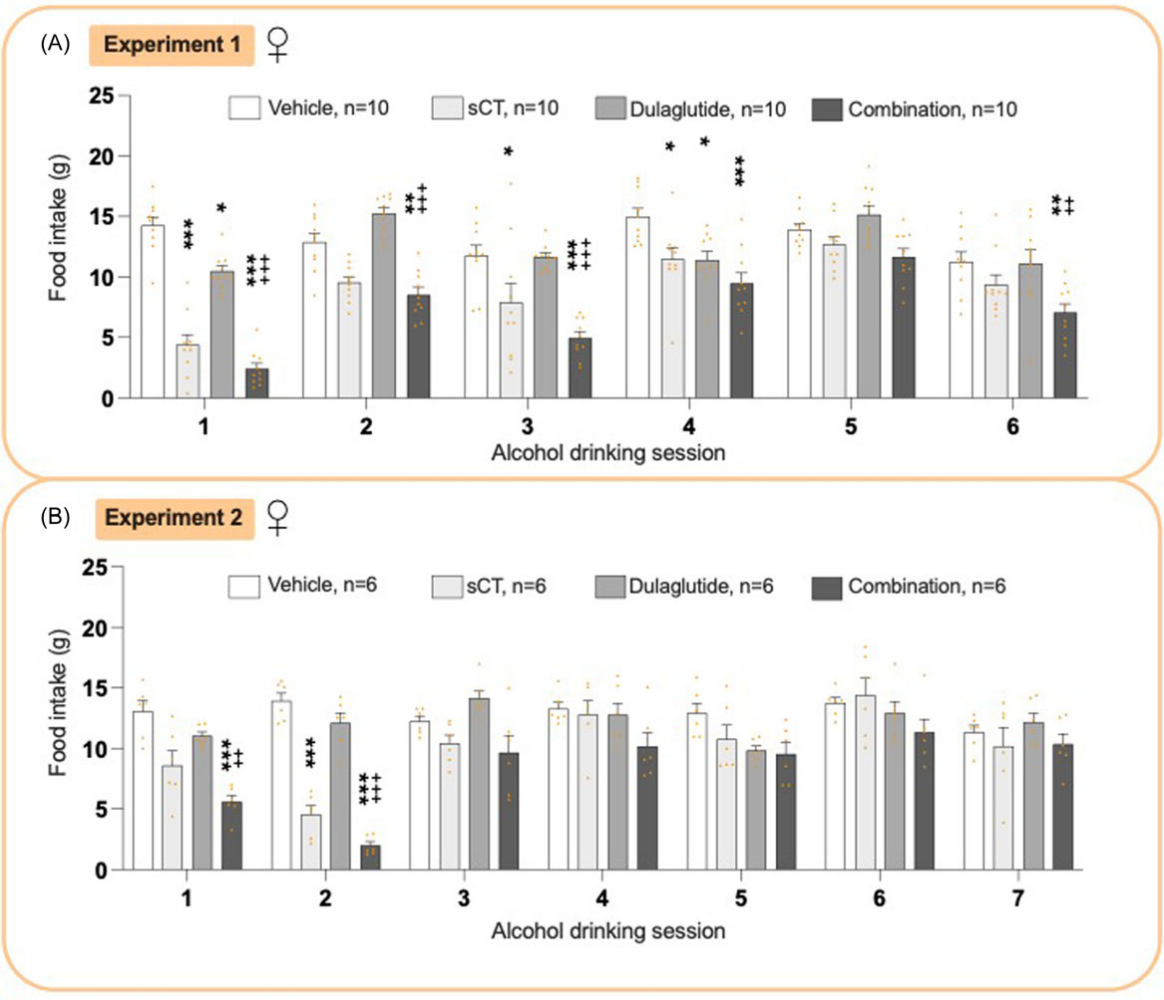
**Treatment effect on food intake in female rats**. (A) In experiment 1, the treatment combination lowered food intake compared to either vehicle (alcohol drinking session 1, 2, 3, 4, 6) or dulaglutide (alcohol drinking session 1, 2, 3). In comparison with vehicle, salmon calcitonin (sCT) (alcohol drinking session 1, 2, 3) and dulaglutide (alcohol drinking session 1) reduced food intake. (B) In experiment 2, the combination treatment decreased food intake compared to vehicle (alcohol drinking session 1, 2) and dulaglutide (alcohol drinking session 1, 2). The monotherapy of sCT reduced food intake compared to vehicle (alcohol drinking session 1). Both in experiment 1 and 2, the combination treatment reduced food intake at more sessions than the monotherapies. Data are presented as mean ± SEM, significant data are illustrated by *P < 0.05, **P < 0.01, ***<0.0001 compared to vehicle, ++P < 0.01, +++P < 0.0001 compared to dulaglutide, #P < 0.05 compared to sCT.

In female rats of experiment 1, treatment led to an overall reduction in food intake (*P*< 0.0001 two-way ANOVA, Fig. 4A, Supplementary Table 2). Compared to vehicle, the combination treatment decreased food intake at sessions 1 (*P*< 0.001, 2 *P* < 0.01, 3-4 *P* < 0.001, 6 (*P*< 0.01). It was also lower than dulaglutide-treated rats at sessions 1–3 (*P*< 0.001), and 6 (*P*< 0.01). Dulaglutide reduced food intake at sessions 1 and 4 (*P*< 0.05), and sCT decreased feeding at sessions 1 (*P*< 0.001, 3–4 *P* < 0.05).

Similarly, in experiment 2, treatment overall reduced food intake (*P*< 0.0001 two-way ANOVA, Fig. 4B, Supplementary Table 2). The combination treatment decreased feeding compared to either vehicle (sessions 1–2, *P* < 0.001) or dulaglutide (sessions 1, *P* < 0.01 and 2, *P* < 0.0001). Notable, sCT lowered the food intake in session 2 (*P*< 0.0001), whereas dulaglutide had no significant effect.

### Effects on body weight in male rats from experiments 1 and 2

**Figure 5.**
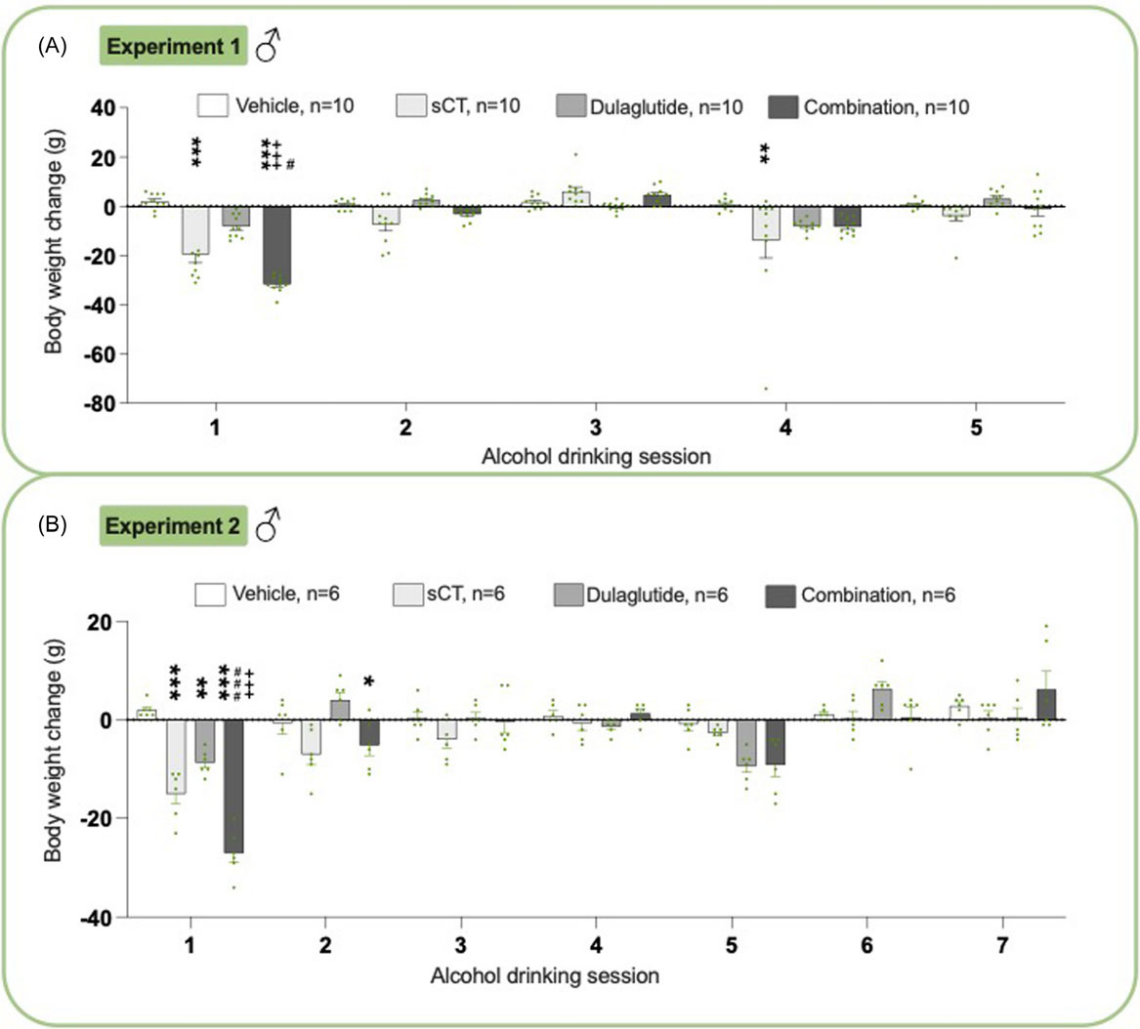
**Treatment effect on body weight in male rats**. (A) At the first alcohol drinking session the combination treatment according to experiment 1 decreased the body weight compared to vehicle, salmon calcitonin (sCT), and dulaglutide treatment. When administered individually, sCT also exhibited a reduction in body weight compared to vehicle (alcohol drinking session 1, 4). Dulaglutide did not alter the body weight. (B) In experiment 2, the combination treatment decreased the body weight compared to vehicle (alcohol drinking session 1, 2), sCT (alcohol drinking session 1), and dulaglutide (alcohol drinking session 1). At the first session, both sCT and dulaglutide alone decreased the body weight compared to vehicle. In both experiments, the combination treatment showed a robust bodyweight reduction compared to either monotherapy. Data are presented as mean ± SEM, significant data are illustrated by *P < 0.05, **P < 0.01, ***<0.0001 compared to vehicle, +++P < 0.0001 compared to dulaglutide, #P < 0.05, ###<0.0001 compared to sCT.

There was an overall treatment effect on male rats’ body weight in experiment 1 (*P*< 0.0001, two-way ANOVA, Fig. 5A, Supplementary Table 3). The combination therapy decreased body weight at session 1 compared to vehicle (*P*< 0.001) and was more effective than either monotherapy (sCT, *P* < 0.05; dulaglutide, *P* < 0.001) at session 1. sCT reduced body weight at sessions 1 (*P*< 0.001) and 4 (*P*< 0.01) compared to vehicle.

In experiment 2, treatment overall reduced the body weight in males (*P*< 0.0001, two-way ANOVA, Fig. 5B, Supplementary Table 3). The combination therapy lowered body weight at sessions 1 (*P*< 0.001) and 2 (*P*< 0.05) compared to vehicle, and was more effective than monotherapies (sCT, *P* < 0.001 and dulaglutide, *P* < 0.001). sCT (*P*< 0.001) and dulaglutide (*P*< 0.01) reduced body weight compared to vehicle. Body weight throughout the experiment is shown in Supplementary Figure 13A.

### Effects on body weight in female rats from experiments 1 and 2

**Figure 6.**
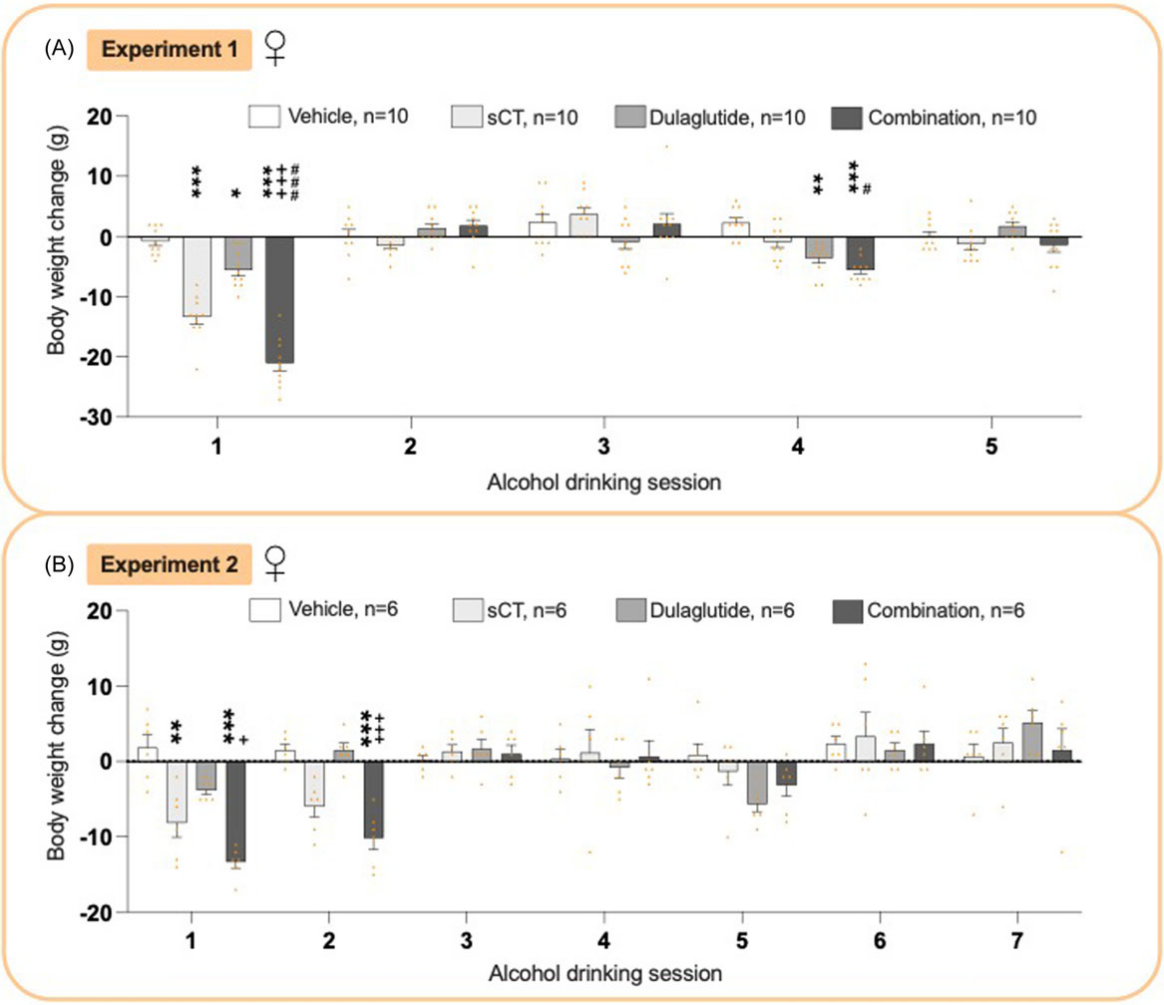
**Treatment effect on body weight change in female rats**. (A) In experiment 1, the combination treatment decreased the body weight compared to vehicle (alcohol drinking session 1, 4), salmon calcitonin (sCT) (alcohol drinking session 1, 4), and dulaglutide (alcohol drinking session 1). Compared to vehicle, sCT (alcohol drinking session 1) and dulaglutide (alcohol drinking session 1, 4) reduced the body weight. (B) In experiment 2, the combination treatment decreased the body weight compared to vehicle (alcohol drinking session 1, 2) and dulaglutide (alcohol drinking session 1, 2). At the first alcohol drinking session, sCT alone decreased the body weight compared to vehicle. The combination treatment showed a more robust decline in body weight, compared to dulaglutide (in both experiments) and sCT (in experiment 1). Data are presented as mean ± SEM, significant data are illustrated by *P < 0.05, **P < 0.01, ***<0.0001 compared to vehicle, +P < 0.05, +++P < 0.0001 compared to dulaglutide, #P < 0.05, ###<0.0001 compared to sCT.

In experiment 1, female rats showed an overall treatment effect on body weight (*P*< 0.0001, two-way ANOVA, Fig. 6A, Supplementary Table 3). The combination treatment decreased body weight compared to vehicle at sessions 1 and 4 (P < 0.001) and was more effective than either sCT (sessions 1, *P* < 0.001 and 4, *P* < 0.05) and dulaglutide (session 1, *P* < 0.001). In comparison to vehicle, dulaglutide (session 1 *P* < 0.05) and sCT (sessions 1 *P* < 0.01 and 4 *P* < 0.01) decreased the body weight.

In experiment 2, treatment overall reduced body weight in females (*P*= 0.0008, two-way ANOVA, Fig. 6B, Supplementary Table 3). The combination treatment lowered body weight compared vehicle (sessions 1–2, *P* < 0.001) and dulaglutide (sessions 1 *P* < 0.05, 2 *P* < 0.001). sCT reduced body weight at session 1 (*P*< 0.01), while dulaglutide had no impact. Body weight throughout the experiment is shown in Supplementary Figure 13B.

### Effects on alcohol preference, water, total fluid, and caloric intake in male rats from experiments 1 and 2

In male rats from Experiment 1, the combination treatment reduced the alcohol preference, whereas neither monotherapy had an effect (Supplementary Figure 3A, I). The combination therapy increased water intake, as did sCT (Supplementary Figure 3B, I). Additionally, sCT increased total fluid intake (Supplementary Figure 3C, I). Moreover, the combination treatment, as well as the monotherapy with either sCT or dulaglutide decreased caloric intake (Supplementary Figure 3D, I).

In experiment 2 of males, treatments did not affect alcohol preference, but sCT increased water intake (session 2, *P* < 0.05). There was an overall treatment increase in the total fluid intake (Supplementary Figure 3E-G, I). Compared to vehicle, or either monotherapy, the combination decreased the caloric intake (Supplementary Figure 3H, I).

### Effects on alcohol preference, water, and total fluid intake in female rats from experiments 1 and 2

In female rats from experiment 1, the combination treatment, sCT and dulaglutide as monotherapy reduced the alcohol preference (Supplementary Figure 4A, G). The combination and sCT enhanced the water intake and total fluid intake (Supplementary Figure 4B-C, G). Moreover, the combination treatment decreased caloric intake compared to vehicle, sCT, or dulaglutide, and both monotherapies reduced caloric intake (Supplementary Figure 3D, I).

In experiment 2, female rats displayed an overall reduction in alcohol preference, without a specific treatment effect (Supplementary Figure 4E, I). The combination treatment and sCT increased the water intake (Supplementary Figure 4F, I), and the total fluid intake (Supplementary Figure 4G, I). Compared to either vehicle or dulaglutide alone, the combination treatment led to lower caloric intake, and sCT also decreased caloric intake (Supplementary Figure 3D, I).

### Effects on gene expression in reward-related brain areas, weight and gene expression in fat tissues, and peptide levels in serum from male and female rats in experiment 1

In experiment 1, the combination reduced alcohol and food intake as well as body weight. To explore tentative underlying mechanisms, gene expression of *GLP-1R* and *AMYR1* (CTR and RAMP1 composing the AMYR1) was assessed in reward-related brain areas (Supplementary Figure 5, *n* = 7–10). In males, treatment did not alter the investigated genes within the PVT, NAcS, or VTA though there was an overall decrease in *RAMP1* expression in LDTg (Supplementary Figure 5A-L, Y). In females, treatment did not affect gene expression in the PVT and NAcS, or most genes in the VTA and LDTg though there was an overall decline in the *GLP-1R* gene expression within the VTA and the *CTR* and *RAMP1* expression in LDTg. Moreover, the combination treatment reduced the expression of *CTR* compared to both vehicle and sCT. Compared to sCT the combination lowered expression of *RAMP1* in this area (Supplementary Figure 5M-X, Z).

The combination therapy in experiment 1 reduced body weight in both male and female rats, but this was not due to reduced fat tissue weight, as no significant effects were found on subcutaneous, gonadal, brown adipose, or renal fat tissue (Supplementary Figure 6A-L).

Neither was the reduction in body weight explained by a treatment effect on the expression of genes important for fat tissue metabolism, (for review, see (Smith and Horwitz, 1969; Liang and Ward, 2006, Seale *et al*., 2008, Bargut *et al*., 2017, Richard *et al*., 2017, Ikeda and Yamada, 2020)), as treatment did not alter the expression of *PGc1α PRDM16,* nor *PPARγ* in brown adipose, subcutaneous, and gonadal fat tissues (Supplementary Figure 7A-X). A trend was observed toward reduced brown adipose tissue weight and increased *UCP1* expression in males (Supplementary Figure 6C, 7D)

Peptide levels in serum were measured to investigate potential mechanisms underlying the treatment’s ability to reduce consummatory behaviour in Experiment 1. In males, there was no treatment effect on corticosterone, any of the measured gut-brain peptides, or cytokines (Supplementary Figure 8). In female rats, there was an overall treatment effect on IL-1β and RANTES, whereas this was not attributed to any specific treatment. No other treatment effects were observed on any other examined peptides (Supplementary Figure 9).

### Effects on fat tissue and organ weight, and histology of the fat tissues in rats of both sexes in experiment 2

In experiment 2, all treatments lowered the body weight in male rats, with the combination treatment showing a greater reduction than either monotherapy. This reduction may be due to an overall reduction in total fat tissue weight in both male and female rats (Supplementary Figure 10A, *K*, U–V).

**Figure 7.**
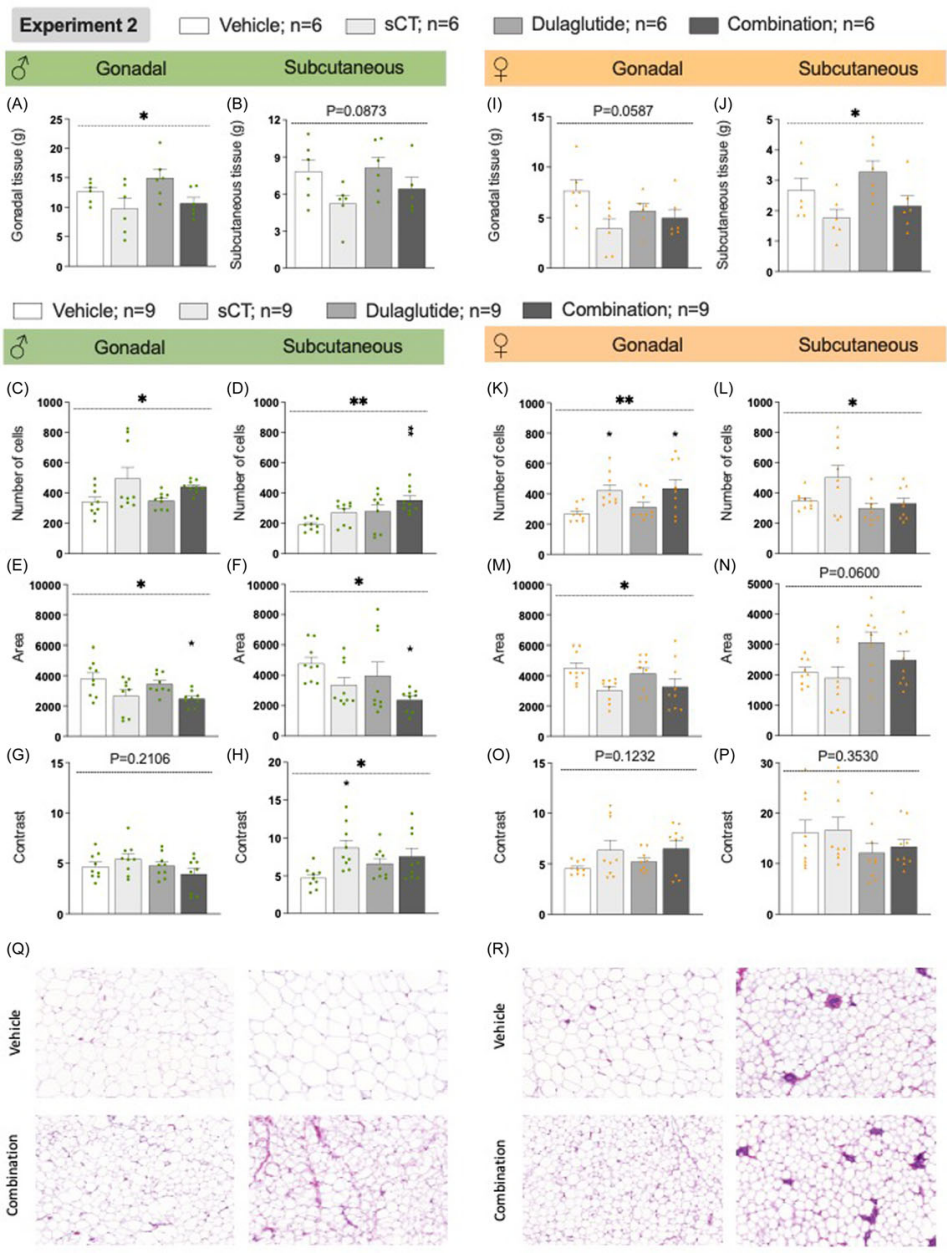
**Treatment effects on gonadal and subcutaneous fat from rats from experiment 2** Effects of experiment 2 on male gonadal (A, C, E, G, Q-the two pictures to the left), male subcutaneous (B, D, F, H, Q-the two pictures to the right), female gonadal (I, K, M, O, R-the two pictures to the left), female subcutaneous (J, L, N, P, R-the two pictures to the right) fat tissues. (A) There was an overall reduction in the weight of gonadal fat tissue in male rats from experiment 2, but this was not attributed to one specific treatment. (B) There was a trend towards a treatment effect on the weight of subcutaneous fat tissue in males. (C) Additional morphological analysis revealed that there was an overall treatment effect on the number of fat cells in gonadal fat tissue, but this increase was not driven by one single treatment. (D) In subcutaneous fat tissue, the combination treatment elevated the number of cells compared to vehicle. Both in (E) gonadal and (F) subcutaneous fat tissue the combination treatment reduced the area of the fat cells, (G-H) whereas the connective tissue (contrast) was unaffected by treatment in both gonadal fat tissue and salmon calcitonin (sCT) as monotherapy increased the contrast in subcutaneous fat tissue. (I) There was a trend towards an overall reduction in the weight of gonadal fat tissue in female rats from experiment 2. (J) Moreover, there was an overall treatment decrease in the weight of the subcutaneous fat tissue in females. However, this treatment reduction was not attributed to one specific treatment. (K) Treatment caused an overall increase in the number of fat cells in gonadal fat tissue, and the increase was evident both after sCT and the combination treatment. (L) In subcutaneous fat tissue, there was an overall increase in the number of cells, but not significantly affected by any treatment. (M) There was an overall decline by treatment in fat cell area in gonadal fat tissue. (N) When it comes to the area of the fat cells in subcutaneous fat tissue, there was a trend toward an overall treatment effect. (O-P) The connective tissue (contrast) was unaffected by treatment in both gonadal and subcutaneous fat tissue. (Q) Representative pictures of gonadal (the two pictures to the left) and subcutaneous (the two pictures to the right) fat tissue after vehicle (top) and combination (bottom) treatment in male rats. (R) Representative pictures from female gonadal (the two pictures to the left) and subcutaneous (the two pictures to the right) fat tissue after vehicle (top) and combination (bottom) treatment. Data are presented as mean ± SEM, significant data are illustrated by *P < 0.05, **P < 0.01 compared to vehicle.

In male rats, treatment reduced gonadal fat weight (*P*= 0.0476, one-way ANOVA) and a tendency to reduce subcutaneous fat (*P*= 0.0873, one-way ANOVA, Fig. 7A-B, Supplementary table 4). Treatment increased the number of fat cells in both gonadal (P = 0.0405, one-way ANOVA) and subcutaneous depots (*P*= 0.0054, one-way ANOVA, Fig. 7C-D, Supplementary table 4) with the combination treatment significantly increasing fat cell count in subcutaneous fat tissues (*P*= 0.0023). The treatment also overall decreased fat cell area in both tissues (gonadal, *P* = 0.0168; subcutaneous *P* = 0.0354, one-way ANOVA, Fig. 7E-F, Supplementary table 4). In both fat tissues (*P*= 0.0297 and *P* = 0.0250 respectively), the combination treatment reduced the area of cells compared to vehicle. Additionally, sCT increased connective tissue in subcutaneous fat (*P*= 0.0157, one-way ANOVA, Fig. 8G-H, Supplementary table 4). This observation is visually represented by illustrative images (Fig. 7Q) and by histograms (Supplementary Figures 11 and 12). A trend toward reduced retroperitoneal fat was noted, while peritoneal and brown adipose tissue weight was unaffected (Supplementary Figure 10B-D, U).

In female rats in Experiment 2, there was a trend toward reduced gonadal fat (*P*= 0.0587, one-way ANOVA) and a significant reduction in subcutaneous fat (*P*= 0.0346, one-way ANOVA, Fig. 7I-J, Supplementary Table 4). The treatment increased fat cell number in both depots (gonadal, *P* = 0.0085; subcutaneous, *P* = 0.0182, one-way ANOVA, Fig. 7K-L, Supplementary table 4), with the combination therapy (*P*= 0.0195) and sCT (*P*= 0.0363) specifically increasing fat cells in gonadal tissue. Treatment reduced fat cell area in gonadal tissue (*P*= 0.0414, one-way ANOVA) and showed a trend in subcutaneous tissue (*P*= 0.0600, one-way ANOVA, Fig. 8M-N, Supplementary Table 4). There was no overall effect on connective tissue in females (Fig. 7O-P, Supplementary Table 4). This observation is visually represented by illustrative images (Fig. 7R) and by histograms (Supplementary Figures 11 and 12). Additionally, there was a trend towards an overall reduction of the weight of retroperitoneal fat tissues, an overall decrease in peritoneal fat, and no effect on the brown adipose tissue weight (Supplementary Figure 10L-N, V).

The reduced body weight observed in experiment 2 was not due to a treatment effect on the rat’s length or the weight of the calf muscle or liver in males (Supplementary Figure 10E-G) or females (Supplementary Figure 10O-Q).

The findings that the treatment did not alter the weights of the spleen or pancreas in male or female rats (Supplementary Figure 10H-I, R-S, U-V), crudely indicate that treatment did not induce inflammation (Esser *et al*., 2014, Mollaei *et al*., 2020). Besides, the blood glucose levels were unaffected by treatment in both sexes excluding this as a confounding factor (Supplementary Figure 10J, T, U-V).

## Discussion

While previous studies found that GLP-1R or AMYR agonists reduce alcohol intake (Kalafateli *et al*., 2019, Marty *et al*., 2020, Vallöf *et al*., 2020, Kalafateli *et al*., 2021, Aranäs *et al*., 2023), we here show that the combination of dulaglutide and sCT reduces alcohol consumption in male and female rats. Specifically, when adding sCT to an ongoing dulaglutide treatment, a reduction in both alcohol intake and preference at several sessions was observed in both male and female rats. Moreover, the combination treatment caused an initial reduction in alcohol intake in both male and female rats when the drugs were injected simultaneously. While the present study did not establish that the combination treatment synergistically decreased alcohol intake, it should be noted that the combination treatment was statistically significantly more effective than sCT at one session. Moreover, combination therapy reduced alcohol intake in a greater number of sessions compared to monotherapies, which may suggest a tendency toward a stronger effect. Another indication of the combination therapy’s greater effect compared to monotherapies was that it reduces alcohol preference in male rats, whereas neither monotherapy alone altered this parameter. In female rats, combination therapy as well as monotherapy decreased the preference for alcohol. A time trend is observed in alcohol preference in male rats in experiment 1, which might be influenced by factors such as sex, strain, age, and experimental conditions (Roman *et al*., 2012, Moore and Lynch, 2015, Lundberg *et al*., 2021). The effect appears to be sex-dependent in this experiment, as female rats in experiment 1 do not exhibit the same trend. To establish synergistic reduction, further studies are needed, potentially using different paradigms, different doses, additional agonists, or other GLP-1R agonists such as semaglutide and thereby assess the optimal effects of this combination therapy on alcohol intake. Although these data are the first demonstrating that the combination of GLP-1R and AMYR agonists lowers alcohol intake, previous studies revealed that dual activation of GLP-1R and AMYR synergistically reduced feeding and body weight in obese rats (Trevaskis *et al*., 2013, Liberini *et al*., 2019, Larsen *et al*., 2021) and decreased food intake in primates (Bello *et al*., 2010). They are in further accordance with alcohol studies using other treatment combinations (Nicholson *et al*., 2018, Zhou *et al*., 2019, Söderpalm *et al*., 2020, Ray *et al*., 2021). It should however be noted that a tolerance towards the ability of the treatment to reduce alcohol intake is observed when the drugs are injected simultaneously. Conversely, tolerance is not observed when sCT is added to an existing dulaglutide treatment, as the combination reduces alcohol intake at several sessions. On a similar note, the ability of a combination of GLP-1R and AMYR agonists to reduce body weight was more profound when an AMYR agonist was added on top of the GLP-1R agonist compared to a situation when both agonists were added simultaneously (Liberini *et al*., 2019).

While prior research indicates that sCT reduces feeding (Perlow *et al*., 1980, Eiden *et al*., 2002, Bello *et al*., 2008, Kalafateli Vallöf *et al*., 2019), whereas the food-modifying effects of dulaglutide are less pronounced (Burness and Scott, 2015, Vallöf *et al*., 2020, Sanada *et al*., 2021), this study demonstrates a significant reduction in feeding resulting from their combination. Specifically, the decrease in food consumption was lower than either monotherapy in both experiments in both sexes. Support for this effect was provided by a previous study of male rats, in which the food intake was significantly reduced after the combination of liraglutide (a GLP-1R agonist) and sCT compared to either monotherapy (Liberini *et al*., 2019).

Attempts were undertaken to define some tentative mechanisms that may contribute to the ability of the combination treatment to reduce alcohol and food intake. One of these is the expression of AMYR1-related genes in the LDTg, which are impacted by combination treatment in both male and female rats. Supportively, local infusion of sCT or exendin-4 into the LDTg decreased alcohol intake (Vallöf, Kalafateli *et al*., 2019, Kalafateli *et al*., 2021). Although inflammation markers are associated with alcohol drinking (Leclercq *et al*., 2012, Piano, 2017, Ahearn *et al*., 2021) as well as the response of GLP-1 (Rakipovski *et al*., 2018), these do not seem to mediate the obtained results herein. Specifically, there was no difference in inflammation marker in serum when sCT was added to an ongoing dulaglutide treatment. In line with this, the weight of the pancreas or spleen was unaffected when both agonists were added simultaneously. As the plasma levels of GLP-1 and amylin, known to relate to feeding and body weight (Moghadam *et al*., 2017, Zanchi *et al*., 2017, Rahati *et al*., 2022), are unaltered by treatment these are less likely factors to drive the outcome observed herein. In the present study, we observe a treatment effect on fat deposits and histology, where factors like leptin and adiponectin are produced (Scherer *et al*., 1995, Obradovic *et al*., 2021). As they influence consummatory behaviours including alcohol (Kuusisto *et al*., 2012, Nova *et al*., 2019, Mehta *et al*., 2020, Weinland *et al*., 2020), upcoming studies should measure these as possible mechanisms of interest. Stress response is another factor that influences consumption patterns (Sinha *et al*., 2011). However, this appears less likely to impact results in the present study as there are no treatment effects on corticosterone in plasma. Another tentative mechanism that may influence the behavioural outcome of the combination treatment is malaise, a side effect reported following GLP-1R agonists (Kanoski *et al*., 2012) and after sCT in one (Boccia *et al*., 2022), but not all studies (Lutz *et al*., 1995, Lutz *et al*., 2000, Mietlicki- Baase *et al*., 2013). However, an earlier study reveals that dulaglutide induces less nausea than other GLP-1R agonists in a clinical situation (Bettge *et al*., 2017). Moreover, in the present study, the combination treatment increased the water intake, and earlier studies report less malaise at the lower dose range used herein (Mietlicki-Baase *et al*., 2013, Bettge *et al*., 2017). Together, these findings imply that malaise is a less likely confounding factor. However, since GLP-1R agonists and AMYR receptor agonists influence different pathways in the brain related to satiety (Roth *et al*., 2012), they may also affect reward mechanisms in distinct ways. Additionally, the combined activation of GLP-1R and AMYR led to greater neuronal activation in the nucleus tractus solitarius compared to both the vehicle and GLP-1R activation alone (Liberini *et al*., 2019). As this region has been recognised as important for modulating alcohol-related behaviours in rodents before (Vallöf *et al*., 2019), future studies should explore the role of both AMYR and GLP-1R in this area for alcohol response.

The combination treatment successfully reduced the body weight in rats, a finding supported by previous studies with liraglutide/exendin-4 and sCT (Bello *et al*., 2010, Trevaskis *et al*., 2013, Liberini *et al*., 2019, Larsen *et al*., 2021). We can herein for the first time conclude that the combination treatment reduces body weight also in female rats. Specifically, the body weight reduction was evident in comparison to vehicle and either monotherapy, and no tolerance towards treatment was observed. When sCT was added to an ongoing dulaglutide treatment, the combination treatment’s ability to reduce the body weight did not appear to be due to a reduced total weight of fat tissues or altered expression of genes important for fat tissue metabolism (Smith and Horwitz, 1969; Liang and Ward, 2006, Wu *et al*., 2012, Richard *et al*., 2017). When dulaglutide and sCT were added simultaneously, the observed body weight reduction may be due to an overall reduction in total fat tissue weight, including the gonadal, subcutaneous, retroperitoneal, or peritoneal fat tissues. The lowered body weight may also be associated with changed morphology of the fat tissue in both male and female rats as observed herein, this aligns with previous research where weight loss results in decreased size of adipocytes (Löfgren *et al*., 2005, Varady *et al*., 2009). However, in neither male nor female rats, the reduced body weight is not due to a treatment effect on the rat’s length, the weight of calf muscle or liver nor on an effect on connective tissue content in the studied fat deposits. While the body weight reduction by the combination treatment was robust and observed in both sexes, the treatment effect is observed in normal-weight rats. The effect on body weight could be due to the decreased caloric intake observed in both experiments.

The present study is associated with several limitations, and one of these is that rats of regular rather than overweight were used. Future studies should thus explore the impact of the combined treatment on alcohol consumption and body weight in obese rats. Moreover, given that different designs were used for the two alcohol-drinking experiments, the treatment outcome cannot be compared directly between the experiments. In the first experiment, where sCT was added to the ongoing dulaglutide treatment, a possible limitation is that treatment started after one day of baseline drinking. However, due to ethical limitations with single housing, prolonged alcohol exposure before dulaglutide treatment was not possible. As alcohol exposure might influence treatment outcomes additional studies are warranted for the future. Another limitation is that the rats from experiments 1 and 2 were sacrificed at two different time points. However, we anticipated the treatment effects would be long-lasting, and a treatment effect would be expected although the rats were euthanized at 24 or 48 hours after the last injection. Another limitation of the study is the absence of pair-fed animals, which complicates the interpretation of the gene expression and serum findings which might be influenced by different body weights. While the present descriptive findings can be used as an indication, it is unclear whether the changes in gene expression are driven by weight reduction, alcohol intake, or food intake or whether these factors are influenced by the gene expression changes. The gene expression of CTR and RAMP1 is just an indication of the expression of AMYR since RAMP1 can be associated with other receptors, this is a limitation of the study. Future studies should investigate single-cell sequencing. Although several tentative mechanisms of action were studied in the present study, no evident actions were observed. Future studies should explore such tentative mechanisms where factors such as glucose regulation, satiety signalling and further investigation of the effect on the reward system should be explored.

Collectively, these findings show that dual activation of GLP-1R and AMYR reduces alcohol consumption, food intake, and body weight in rats of both sexes. As previous preclinical studies on GLP-1R agonists have been translated into humans, where a reduced alcohol intake was observed in overweight AUD patients treated with these agonists (Klausen *et al*., 2022, Quddos *et al*., 2023, Richards *et al*., 2023; Probst *et al*., 2023; Wium-Andersen *et al*., 2022; Bremmer & Hendershot, 2024), a clinical relevance might be argued. However, given that synergy was not established herein, and tolerance to treatment was observed further preclinical investigations are needed to assess the combined effects on alcohol intake.

## Supporting information

Aranäs et al. supplementary material 1 Aranäs et al. supplementary material

Aranäs et al. supplementary material 2 Aranäs et al. supplementary material

## Data Availability

The data that support the findings of this study are available from the corresponding author upon reasonable request.

## Supplementary material

The supplementary material for this article can be found at https://doi.org/10.1017/neu.2024.58.
