# Supplementary material for: The combination of a glucagon-like peptide-1 and amylin receptor agonists reduces alcohol consumption in both male and female rats
Source: Acta Neuropsychiatr. 2024 Dec 6;37:e42. doi: 10.1017/neu.2024.58 (PMC13130276; doi:10.1017/neu.2024.58)
Supplement: Aranäs et al. supplementary material 1 — Aranäs et al. supplementary material [file S0924270824000589sup001.pptx]

## Slide 1
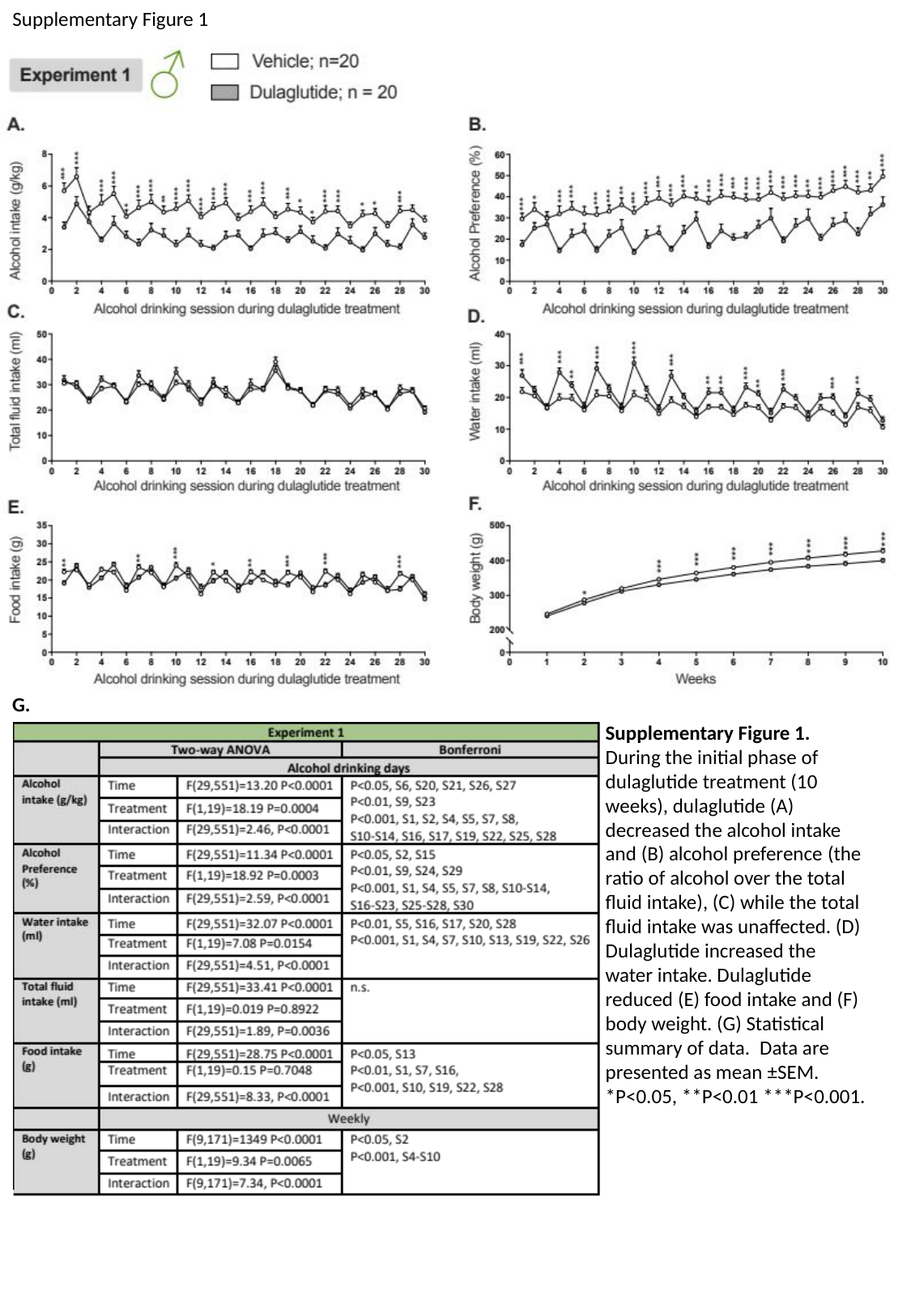

Supplementary Figure 1
G.
Supplementary Figure 1.
During the initial phase of dulaglutide treatment (10 weeks), dulaglutide (A) decreased the alcohol intake and (B) alcohol preference (the ratio of alcohol over the total fluid intake), (C) while the total fluid intake was unaffected. (D) Dulaglutide increased the water intake. Dulaglutide reduced (E) food intake and (F) body weight. (G) Statistical summary of data. Data are presented as mean ±SEM. *P<0.05, **P<0.01 ***P<0.001.

## Slide 2
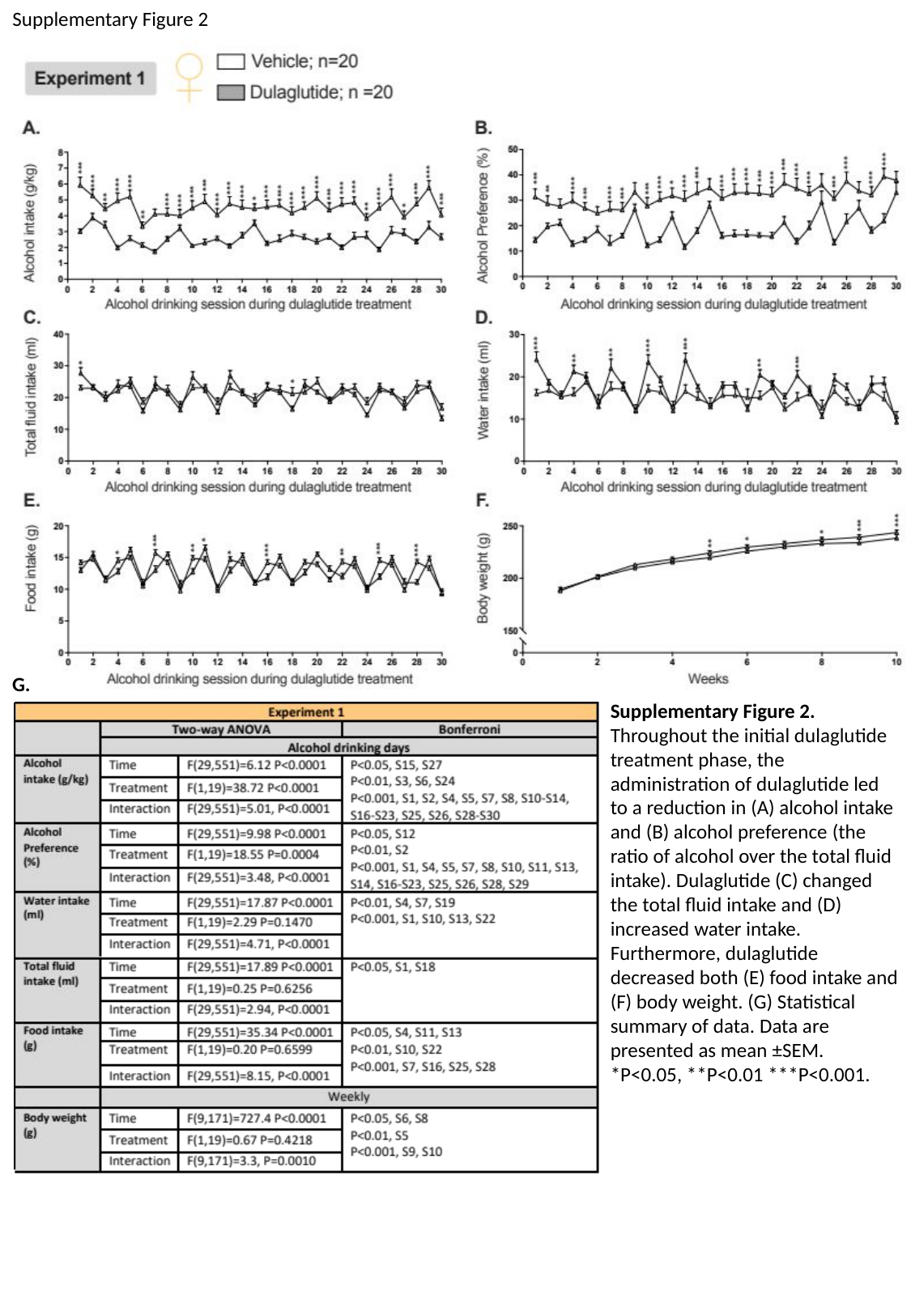

Supplementary Figure 2
G.
Supplementary Figure 2. Throughout the initial dulaglutide treatment phase, the administration of dulaglutide led to a reduction in (A) alcohol intake and (B) alcohol preference (the ratio of alcohol over the total fluid intake). Dulaglutide (C) changed the total fluid intake and (D) increased water intake. Furthermore, dulaglutide decreased both (E) food intake and (F) body weight. (G) Statistical summary of data. Data are presented as mean ±SEM. *P<0.05, **P<0.01 ***P<0.001.

## Slide 3
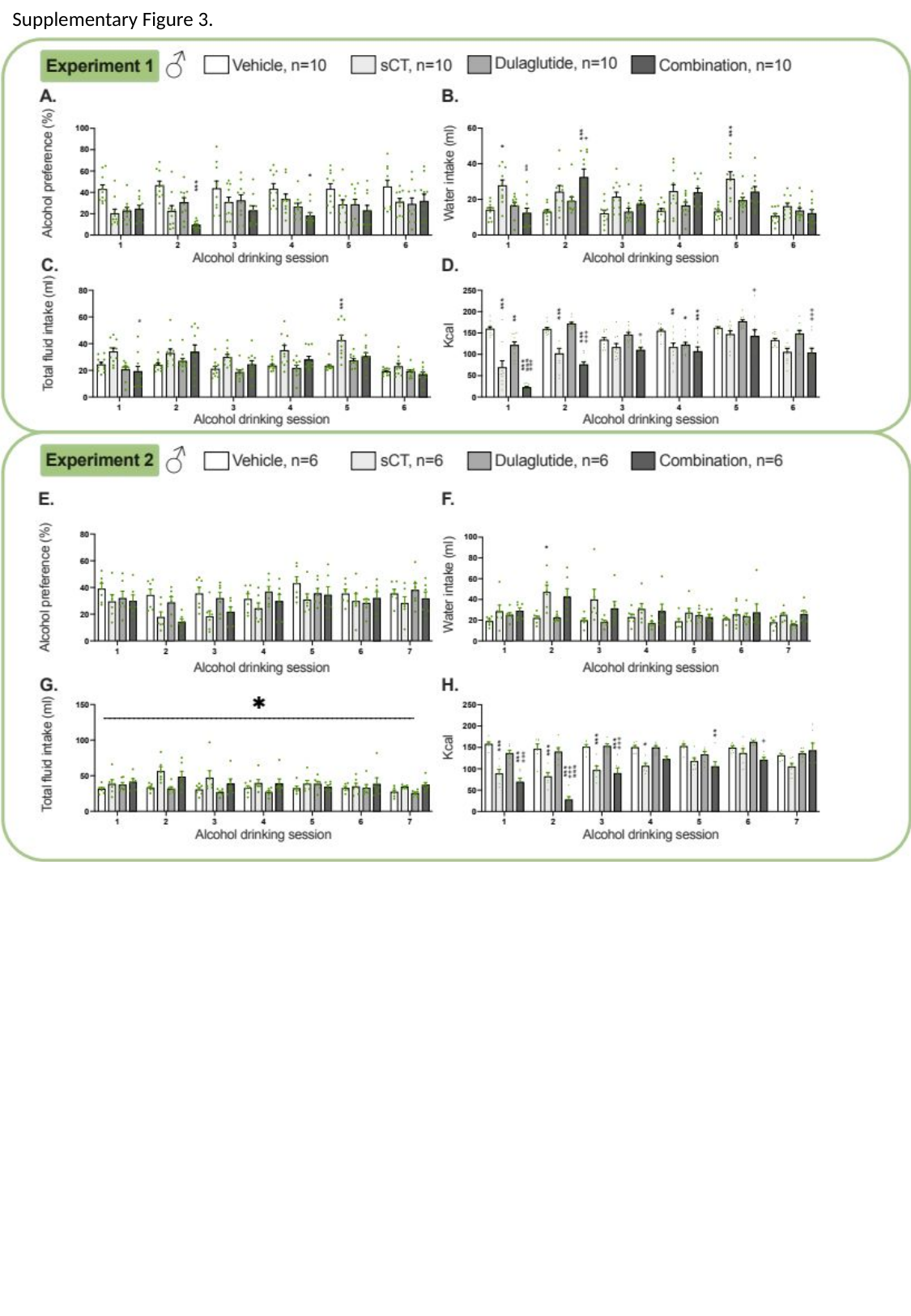

Supplementary Figure 3.

## Slide 4
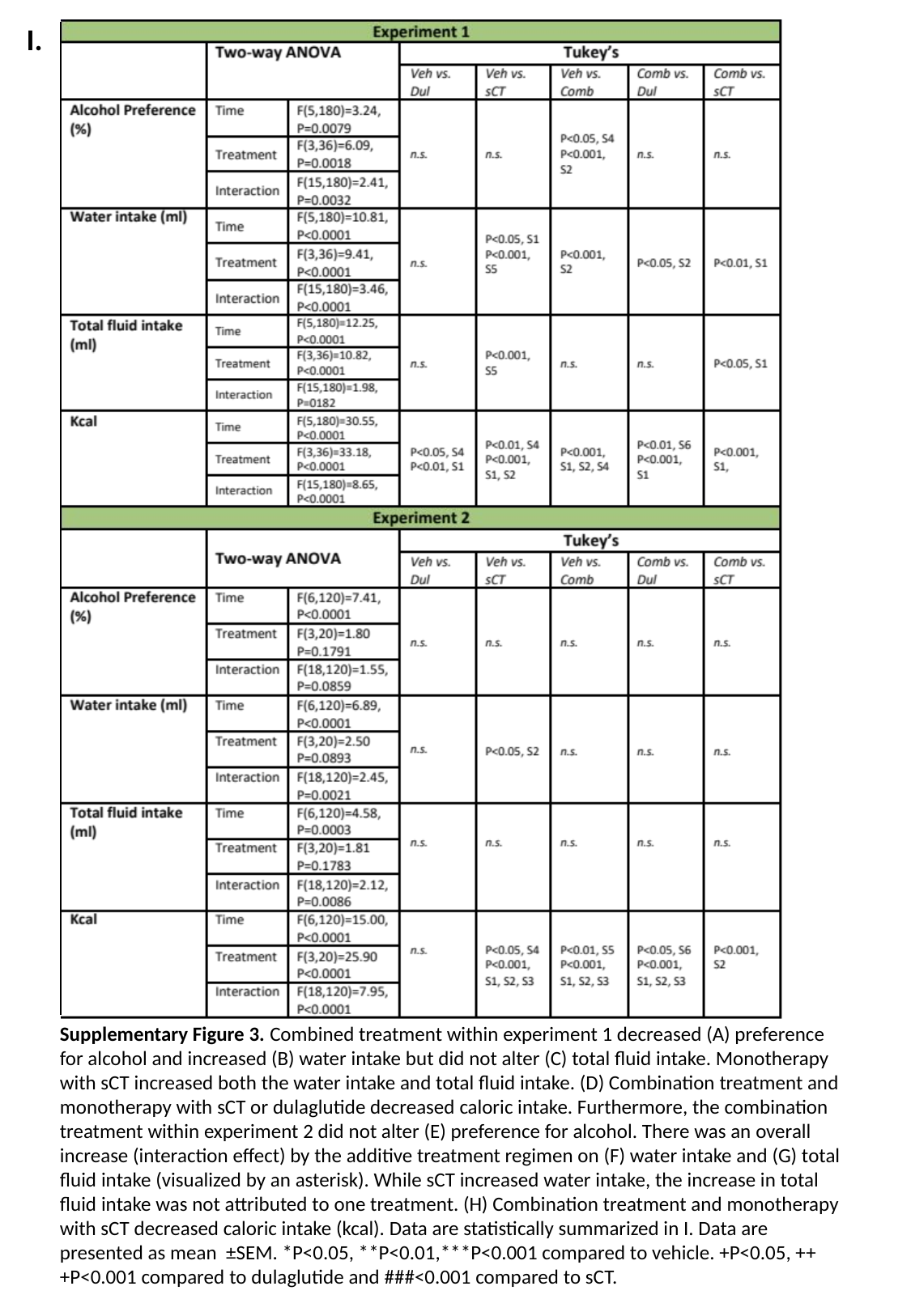

I.
Supplementary Figure 3. Combined treatment within experiment 1 decreased (A) preference for alcohol and increased (B) water intake but did not alter (C) total fluid intake. Monotherapy with sCT increased both the water intake and total fluid intake. (D) Combination treatment and monotherapy with sCT or dulaglutide decreased caloric intake. Furthermore, the combination treatment within experiment 2 did not alter (E) preference for alcohol. There was an overall increase (interaction effect) by the additive treatment regimen on (F) water intake and (G) total fluid intake (visualized by an asterisk). While sCT increased water intake, the increase in total fluid intake was not attributed to one treatment. (H) Combination treatment and monotherapy with sCT decreased caloric intake (kcal). Data are statistically summarized in I. Data are presented as mean ±SEM. *P<0.05, **P<0.01,***P<0.001 compared to vehicle. +P<0.05, +++P<0.001 compared to dulaglutide and ###<0.001 compared to sCT.

## Slide 5
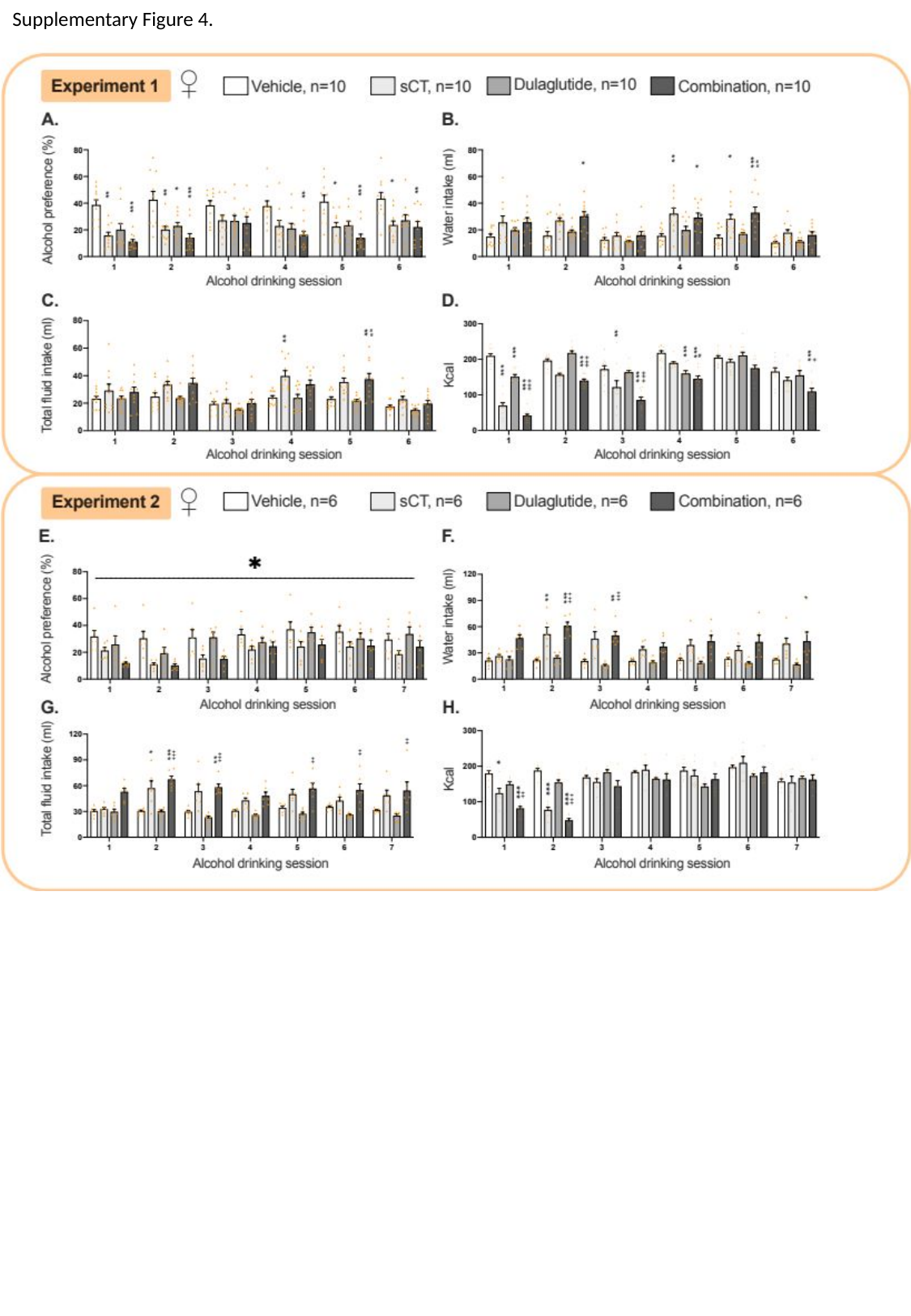

Supplementary Figure 4.

## Slide 6
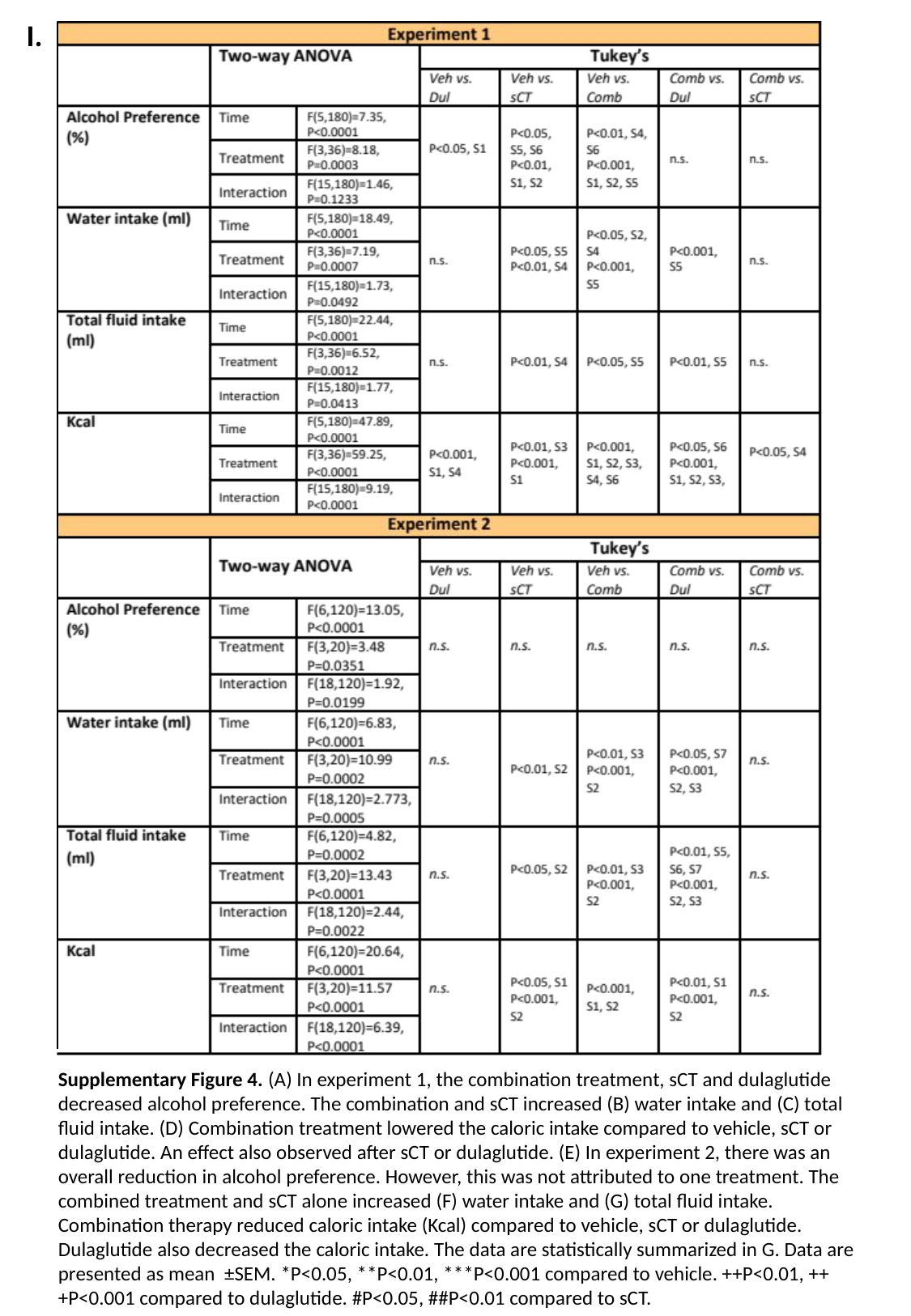

I.
Supplementary Figure 4. (A) In experiment 1, the combination treatment, sCT and dulaglutide decreased alcohol preference. The combination and sCT increased (B) water intake and (C) total fluid intake. (D) Combination treatment lowered the caloric intake compared to vehicle, sCT or dulaglutide. An effect also observed after sCT or dulaglutide. (E) In experiment 2, there was an overall reduction in alcohol preference. However, this was not attributed to one treatment. The combined treatment and sCT alone increased (F) water intake and (G) total fluid intake. Combination therapy reduced caloric intake (Kcal) compared to vehicle, sCT or dulaglutide. Dulaglutide also decreased the caloric intake. The data are statistically summarized in G. Data are presented as mean ±SEM. *P<0.05, **P<0.01, ***P<0.001 compared to vehicle. ++P<0.01, +++P<0.001 compared to dulaglutide. #P<0.05, ##P<0.01 compared to sCT.

## Slide 7
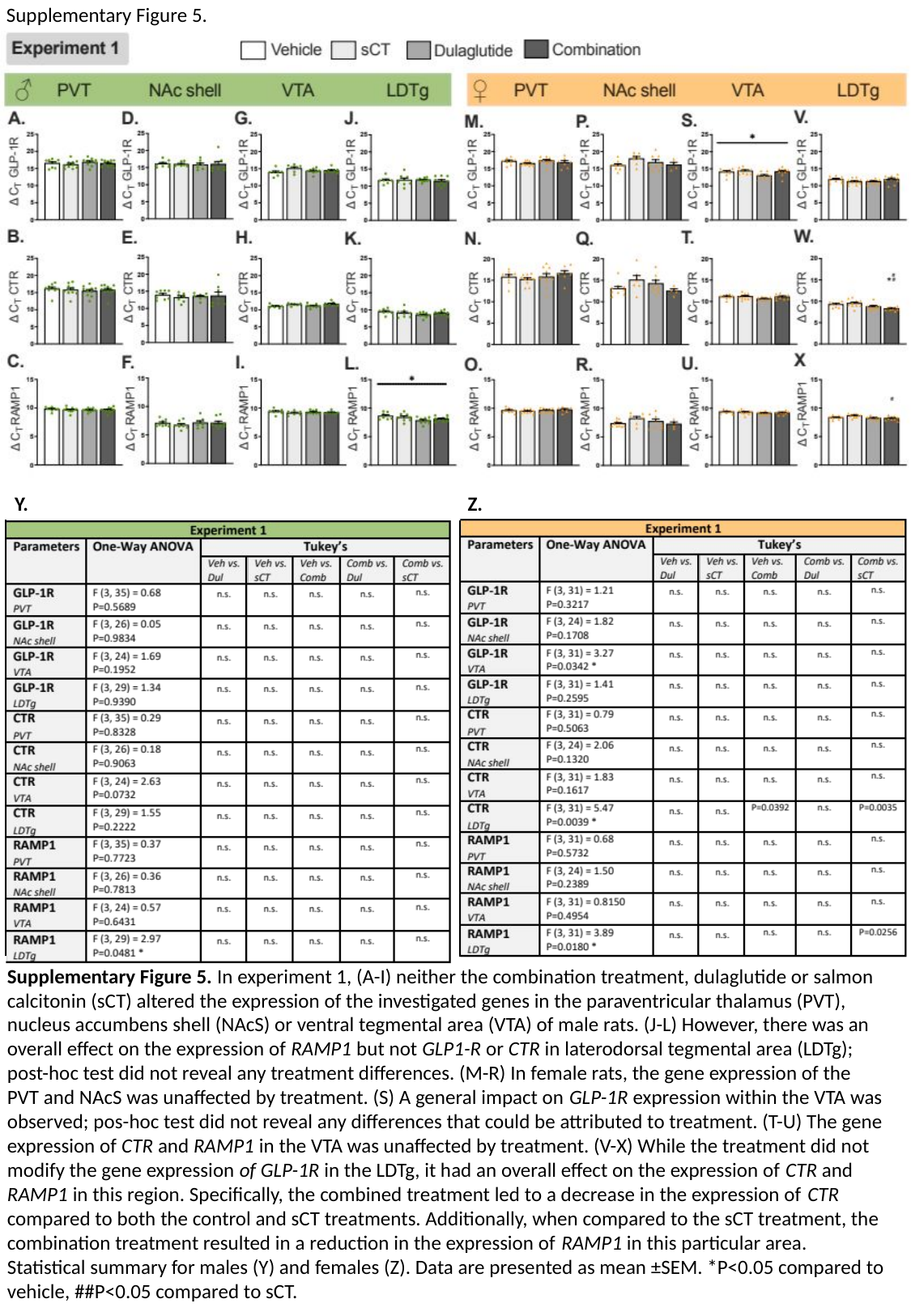

Supplementary Figure 5.
Y.
Z.
Supplementary Figure 5. In experiment 1, (A-I) neither the combination treatment, dulaglutide or salmon calcitonin (sCT) altered the expression of the investigated genes in the paraventricular thalamus (PVT), nucleus accumbens shell (NAcS) or ventral tegmental area (VTA) of male rats. (J-L) However, there was an overall effect on the expression of RAMP1 but not GLP1-R or CTR in laterodorsal tegmental area (LDTg); post-hoc test did not reveal any treatment differences. (M-R) In female rats, the gene expression of the PVT and NAcS was unaffected by treatment. (S) A general impact on GLP-1R expression within the VTA was observed; pos-hoc test did not reveal any differences that could be attributed to treatment. (T-U) The gene expression of CTR and RAMP1 in the VTA was unaffected by treatment. (V-X) While the treatment did not modify the gene expression of GLP-1R in the LDTg, it had an overall effect on the expression of CTR and RAMP1 in this region. Specifically, the combined treatment led to a decrease in the expression of CTR compared to both the control and sCT treatments. Additionally, when compared to the sCT treatment, the combination treatment resulted in a reduction in the expression of RAMP1 in this particular area. Statistical summary for males (Y) and females (Z). Data are presented as mean ±SEM. *P<0.05 compared to vehicle, ##P<0.05 compared to sCT.

## Slide 8
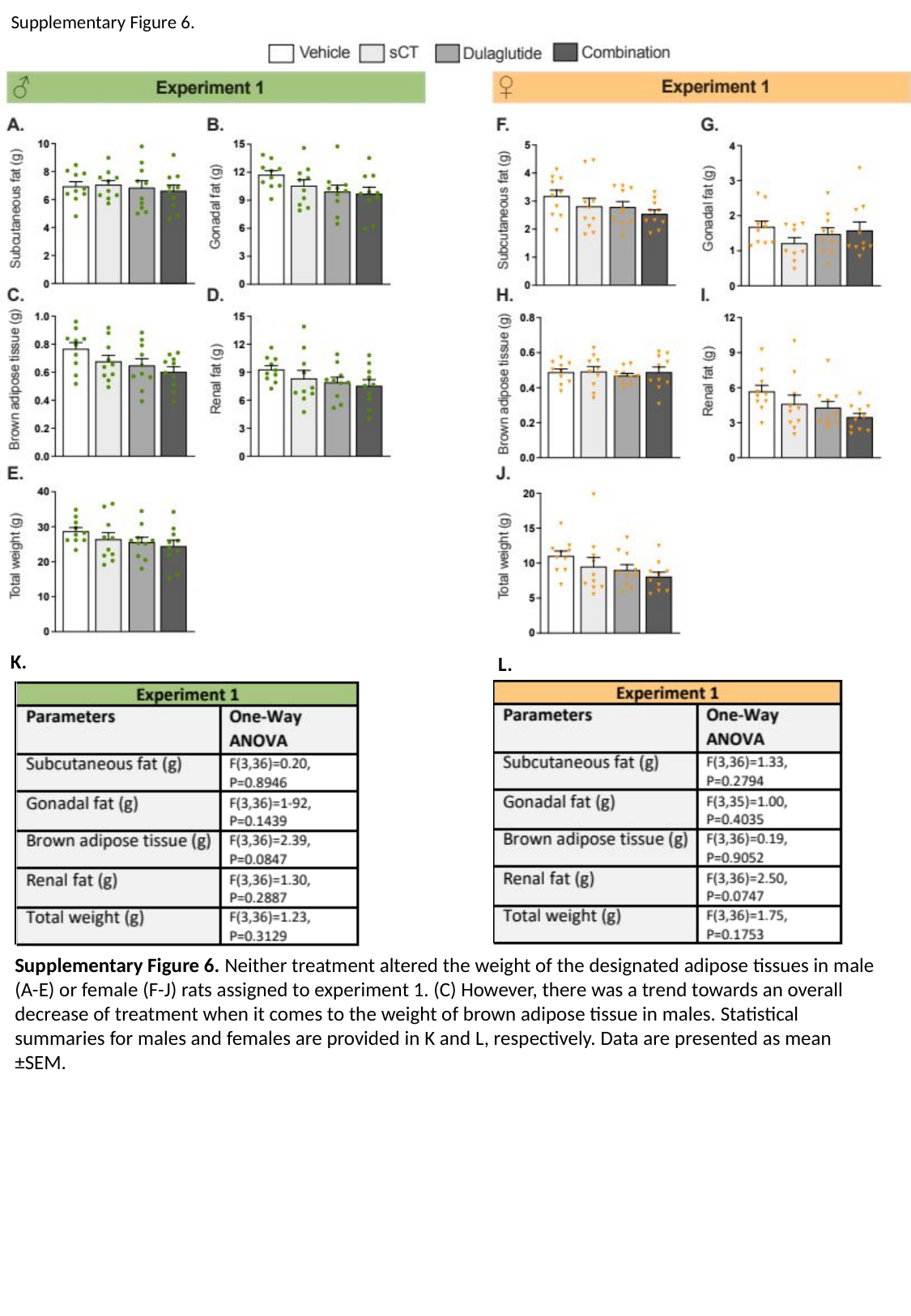

Supplementary Figure 6.
K.
L.
Supplementary Figure 6. Neither treatment altered the weight of the designated adipose tissues in male (A-E) or female (F-J) rats assigned to experiment 1. (C) However, there was a trend towards an overall decrease of treatment when it comes to the weight of brown adipose tissue in males. Statistical summaries for males and females are provided in K and L, respectively. Data are presented as mean ±SEM.

## Slide 9
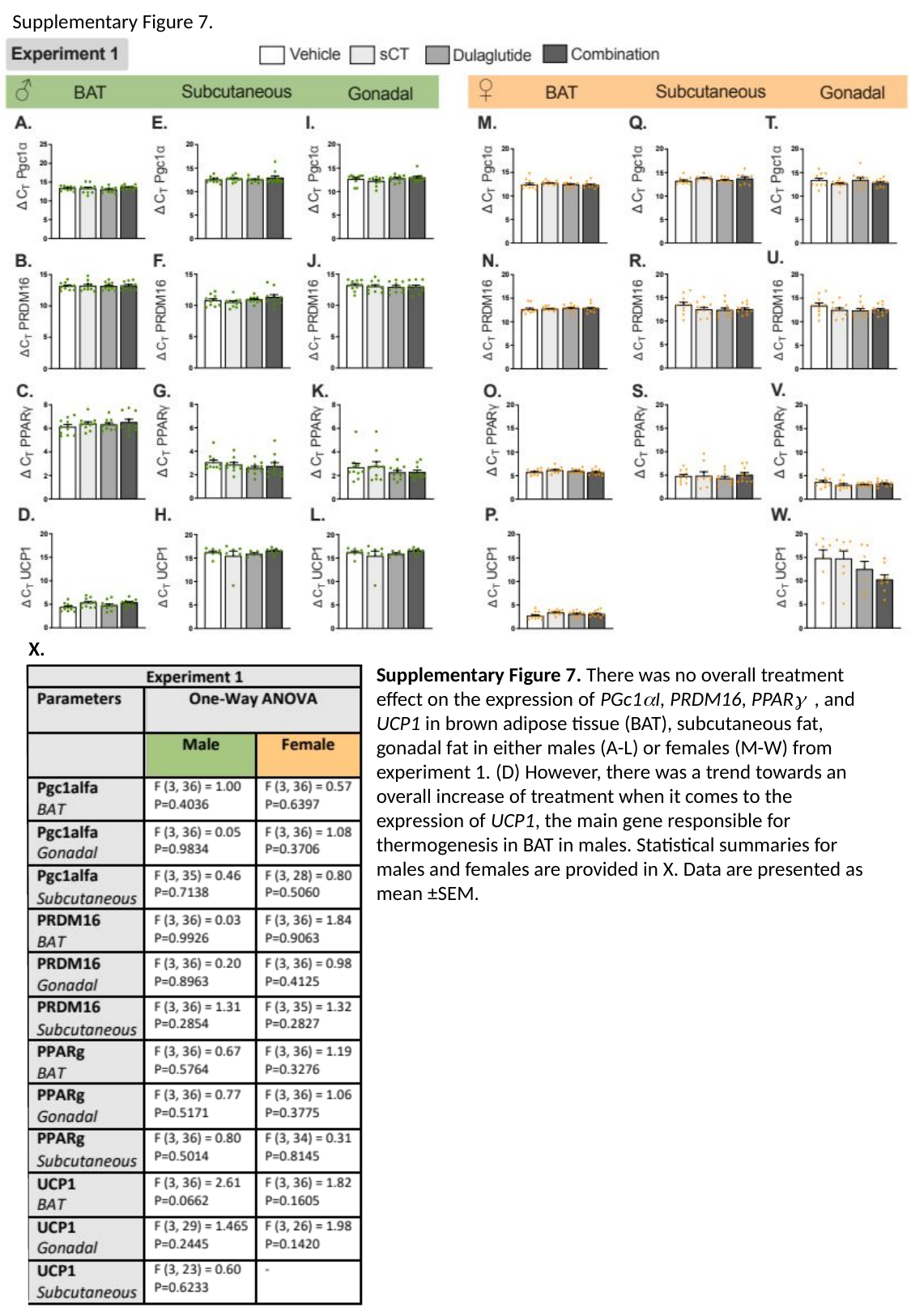

Supplementary Figure 7.
X.
Supplementary Figure 7. There was no overall treatment effect on the expression of PGc1αI, PRDM16, PPARγ , and UCP1 in brown adipose tissue (BAT), subcutaneous fat, gonadal fat in either males (A-L) or females (M-W) from experiment 1. (D) However, there was a trend towards an overall increase of treatment when it comes to the expression of UCP1, the main gene responsible for thermogenesis in BAT in males. Statistical summaries for males and females are provided in X. Data are presented as mean ±SEM.

## Slide 10
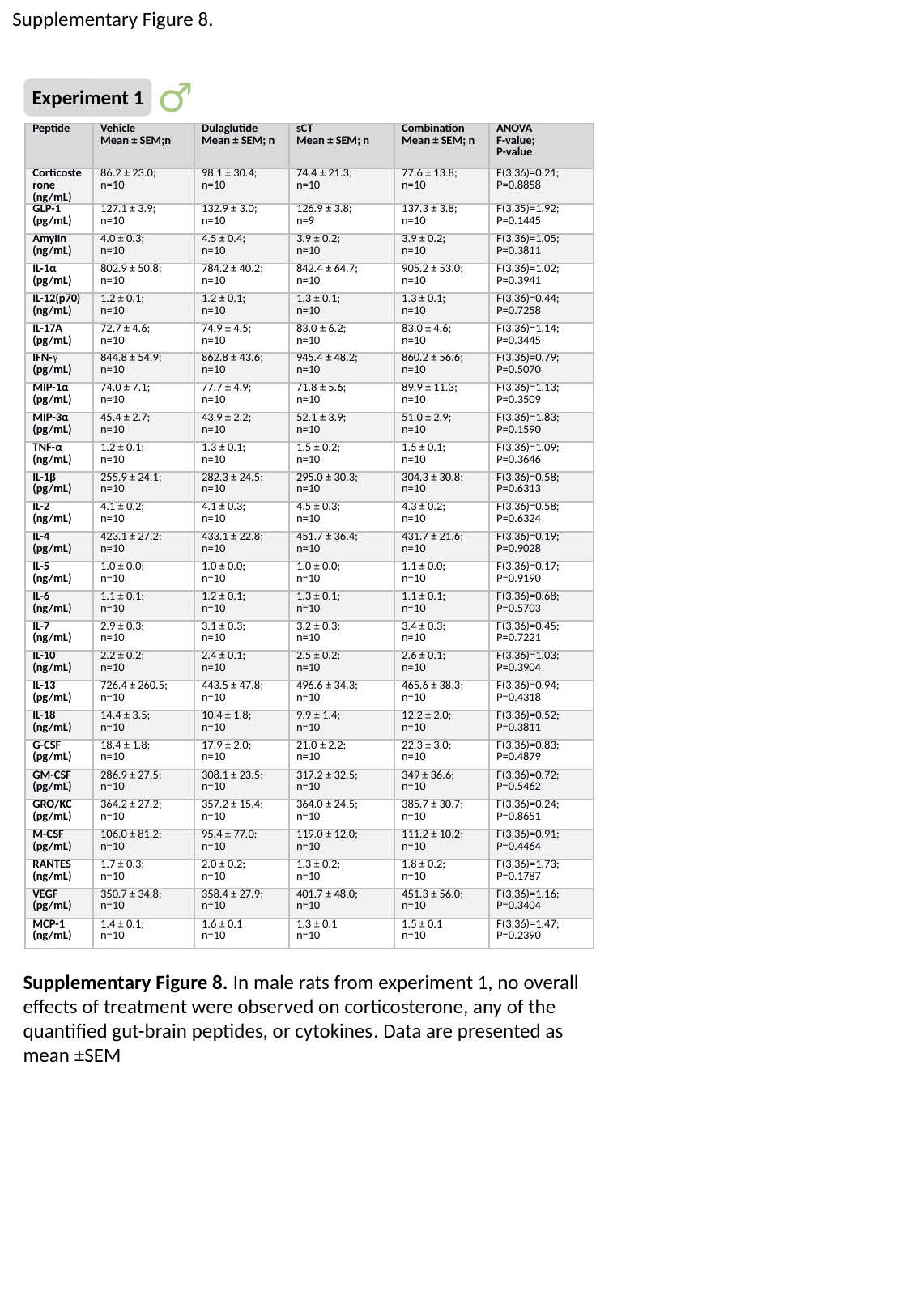

Supplementary Figure 8.
♂
Experiment 1
| Peptide | Vehicle Mean ± SEM;n | Dulaglutide Mean ± SEM; n | sCT Mean ± SEM; n | Combination Mean ± SEM; n | ANOVA F-value; P-value |
| --- | --- | --- | --- | --- | --- |
| Corticosterone (ng/mL) | 86.2 ± 23.0; n=10 | 98.1 ± 30.4; n=10 | 74.4 ± 21.3; n=10 | 77.6 ± 13.8; n=10 | F(3,36)=0.21; P=0.8858 |
| GLP-1 (pg/mL) | 127.1 ± 3.9; n=10 | 132.9 ± 3.0; n=10 | 126.9 ± 3.8; n=9 | 137.3 ± 3.8; n=10 | F(3,35)=1.92; P=0.1445 |
| Amylin (ng/mL) | 4.0 ± 0.3; n=10 | 4.5 ± 0.4; n=10 | 3.9 ± 0.2; n=10 | 3.9 ± 0.2; n=10 | F(3,36)=1.05; P=0.3811 |
| IL-1α (pg/mL) | 802.9 ± 50.8; n=10 | 784.2 ± 40.2; n=10 | 842.4 ± 64.7; n=10 | 905.2 ± 53.0; n=10 | F(3,36)=1.02; P=0.3941 |
| IL-12(p70) (ng/mL) | 1.2 ± 0.1; n=10 | 1.2 ± 0.1; n=10 | 1.3 ± 0.1; n=10 | 1.3 ± 0.1; n=10 | F(3,36)=0.44; P=0.7258 |
| IL-17A (pg/mL) | 72.7 ± 4.6; n=10 | 74.9 ± 4.5; n=10 | 83.0 ± 6.2; n=10 | 83.0 ± 4.6; n=10 | F(3,36)=1.14; P=0.3445 |
| IFN-γ (pg/mL) | 844.8 ± 54.9; n=10 | 862.8 ± 43.6; n=10 | 945.4 ± 48.2; n=10 | 860.2 ± 56.6; n=10 | F(3,36)=0.79; P=0.5070 |
| MIP-1α (pg/mL) | 74.0 ± 7.1; n=10 | 77.7 ± 4.9; n=10 | 71.8 ± 5.6; n=10 | 89.9 ± 11.3; n=10 | F(3,36)=1.13; P=0.3509 |
| MIP-3α (pg/mL) | 45.4 ± 2.7; n=10 | 43.9 ± 2.2; n=10 | 52.1 ± 3.9; n=10 | 51.0 ± 2.9; n=10 | F(3,36)=1.83; P=0.1590 |
| TNF-α (ng/mL) | 1.2 ± 0.1; n=10 | 1.3 ± 0.1; n=10 | 1.5 ± 0.2; n=10 | 1.5 ± 0.1; n=10 | F(3,36)=1.09; P=0.3646 |
| IL-1β (pg/mL) | 255.9 ± 24.1; n=10 | 282.3 ± 24.5; n=10 | 295.0 ± 30.3; n=10 | 304.3 ± 30.8; n=10 | F(3,36)=0.58; P=0.6313 |
| IL-2 (ng/mL) | 4.1 ± 0.2; n=10 | 4.1 ± 0.3; n=10 | 4.5 ± 0.3; n=10 | 4.3 ± 0.2; n=10 | F(3,36)=0.58; P=0.6324 |
| IL-4 (pg/mL) | 423.1 ± 27.2; n=10 | 433.1 ± 22.8; n=10 | 451.7 ± 36.4; n=10 | 431.7 ± 21.6; n=10 | F(3,36)=0.19; P=0.9028 |
| IL-5 (ng/mL) | 1.0 ± 0.0; n=10 | 1.0 ± 0.0; n=10 | 1.0 ± 0.0; n=10 | 1.1 ± 0.0; n=10 | F(3,36)=0.17; P=0.9190 |
| IL-6 (ng/mL) | 1.1 ± 0.1; n=10 | 1.2 ± 0.1; n=10 | 1.3 ± 0.1; n=10 | 1.1 ± 0.1; n=10 | F(3,36)=0.68; P=0.5703 |
| IL-7 (ng/mL) | 2.9 ± 0.3; n=10 | 3.1 ± 0.3; n=10 | 3.2 ± 0.3; n=10 | 3.4 ± 0.3; n=10 | F(3,36)=0.45; P=0.7221 |
| IL-10 (ng/mL) | 2.2 ± 0.2; n=10 | 2.4 ± 0.1; n=10 | 2.5 ± 0.2; n=10 | 2.6 ± 0.1; n=10 | F(3,36)=1.03; P=0.3904 |
| IL-13 (pg/mL) | 726.4 ± 260.5; n=10 | 443.5 ± 47.8; n=10 | 496.6 ± 34.3; n=10 | 465.6 ± 38.3; n=10 | F(3,36)=0.94; P=0.4318 |
| IL-18 (ng/mL) | 14.4 ± 3.5; n=10 | 10.4 ± 1.8; n=10 | 9.9 ± 1.4; n=10 | 12.2 ± 2.0; n=10 | F(3,36)=0.52; P=0.3811 |
| G-CSF (pg/mL) | 18.4 ± 1.8; n=10 | 17.9 ± 2.0; n=10 | 21.0 ± 2.2; n=10 | 22.3 ± 3.0; n=10 | F(3,36)=0.83; P=0.4879 |
| GM-CSF (pg/mL) | 286.9 ± 27.5; n=10 | 308.1 ± 23.5; n=10 | 317.2 ± 32.5; n=10 | 349 ± 36.6; n=10 | F(3,36)=0.72; P=0.5462 |
| GRO/KC (pg/mL) | 364.2 ± 27.2; n=10 | 357.2 ± 15.4; n=10 | 364.0 ± 24.5; n=10 | 385.7 ± 30.7; n=10 | F(3,36)=0.24; P=0.8651 |
| M-CSF (pg/mL) | 106.0 ± 81.2; n=10 | 95.4 ± 77.0; n=10 | 119.0 ± 12.0; n=10 | 111.2 ± 10.2; n=10 | F(3,36)=0.91; P=0.4464 |
| RANTES (ng/mL) | 1.7 ± 0.3; n=10 | 2.0 ± 0.2; n=10 | 1.3 ± 0.2; n=10 | 1.8 ± 0.2; n=10 | F(3,36)=1.73; P=0.1787 |
| VEGF (pg/mL) | 350.7 ± 34.8; n=10 | 358.4 ± 27.9; n=10 | 401.7 ± 48.0; n=10 | 451.3 ± 56.0; n=10 | F(3,36)=1.16; P=0.3404 |
| MCP-1 (ng/mL) | 1.4 ± 0.1; n=10 | 1.6 ± 0.1 n=10 | 1.3 ± 0.1 n=10 | 1.5 ± 0.1 n=10 | F(3,36)=1.47; P=0.2390 |
Supplementary Figure 8. In male rats from experiment 1, no overall effects of treatment were observed on corticosterone, any of the quantified gut-brain peptides, or cytokines. Data are presented as mean ±SEM

## Slide 11
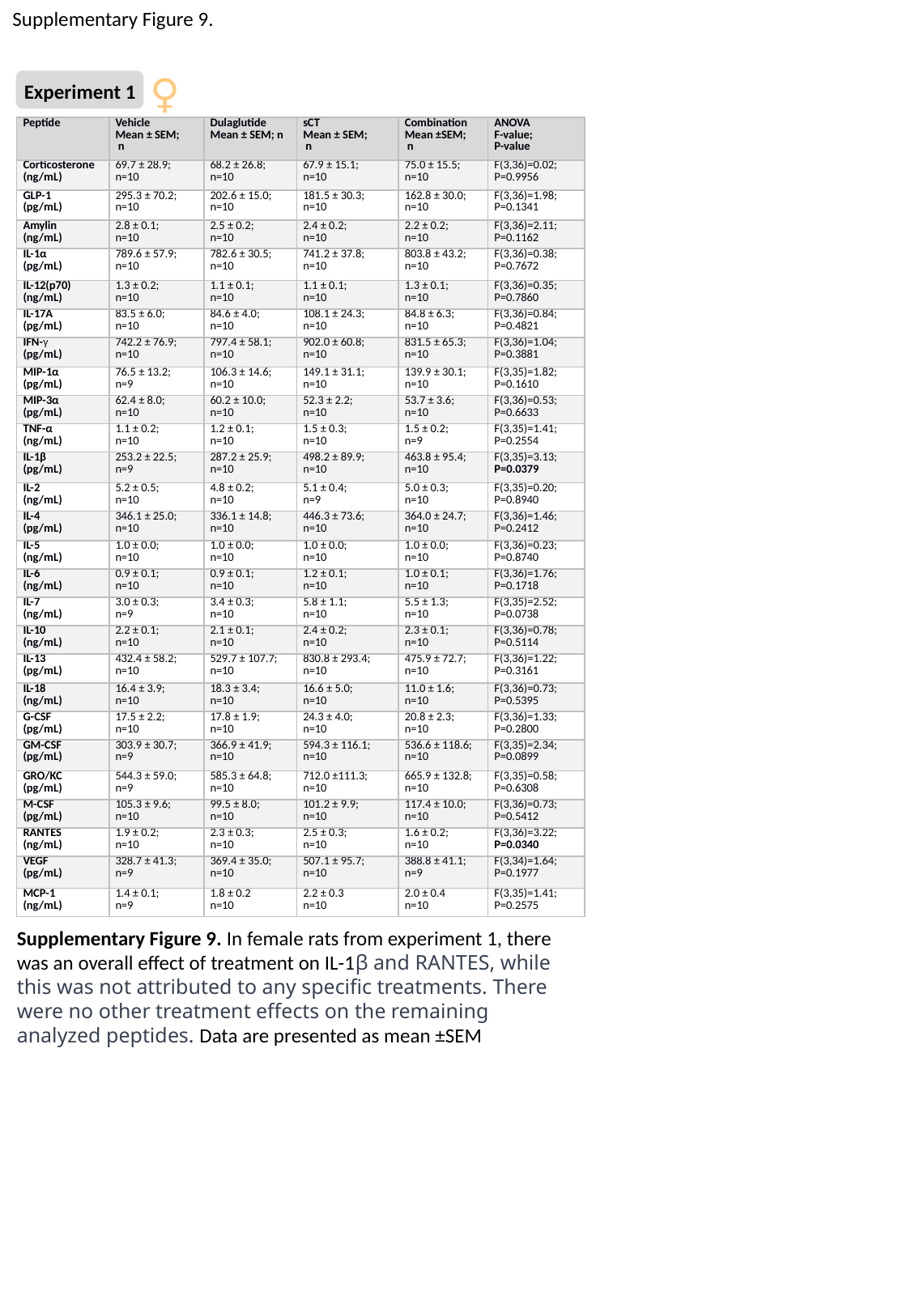

Supplementary Figure 9.
♀
Experiment 1
| Peptide | Vehicle Mean ± SEM; n | Dulaglutide Mean ± SEM; n | sCT Mean ± SEM; n | Combination Mean ±SEM; n | ANOVA F-value; P-value |
| --- | --- | --- | --- | --- | --- |
| Corticosterone (ng/mL) | 69.7 ± 28.9; n=10 | 68.2 ± 26.8; n=10 | 67.9 ± 15.1; n=10 | 75.0 ± 15.5; n=10 | F(3,36)=0.02; P=0.9956 |
| GLP-1 (pg/mL) | 295.3 ± 70.2; n=10 | 202.6 ± 15.0; n=10 | 181.5 ± 30.3; n=10 | 162.8 ± 30.0; n=10 | F(3,36)=1.98; P=0.1341 |
| Amylin (ng/mL) | 2.8 ± 0.1; n=10 | 2.5 ± 0.2; n=10 | 2.4 ± 0.2; n=10 | 2.2 ± 0.2; n=10 | F(3,36)=2.11; P=0.1162 |
| IL-1α (pg/mL) | 789.6 ± 57.9; n=10 | 782.6 ± 30.5; n=10 | 741.2 ± 37.8; n=10 | 803.8 ± 43.2; n=10 | F(3,36)=0.38; P=0.7672 |
| IL-12(p70) (ng/mL) | 1.3 ± 0.2; n=10 | 1.1 ± 0.1; n=10 | 1.1 ± 0.1; n=10 | 1.3 ± 0.1; n=10 | F(3,36)=0.35; P=0.7860 |
| IL-17A (pg/mL) | 83.5 ± 6.0; n=10 | 84.6 ± 4.0; n=10 | 108.1 ± 24.3; n=10 | 84.8 ± 6.3; n=10 | F(3,36)=0.84; P=0.4821 |
| IFN-γ (pg/mL) | 742.2 ± 76.9; n=10 | 797.4 ± 58.1; n=10 | 902.0 ± 60.8; n=10 | 831.5 ± 65.3; n=10 | F(3,36)=1.04; P=0.3881 |
| MIP-1α (pg/mL) | 76.5 ± 13.2; n=9 | 106.3 ± 14.6; n=10 | 149.1 ± 31.1; n=10 | 139.9 ± 30.1; n=10 | F(3,35)=1.82; P=0.1610 |
| MIP-3α (pg/mL) | 62.4 ± 8.0; n=10 | 60.2 ± 10.0; n=10 | 52.3 ± 2.2; n=10 | 53.7 ± 3.6; n=10 | F(3,36)=0.53; P=0.6633 |
| TNF-α (ng/mL) | 1.1 ± 0.2; n=10 | 1.2 ± 0.1; n=10 | 1.5 ± 0.3; n=10 | 1.5 ± 0.2; n=9 | F(3,35)=1.41; P=0.2554 |
| IL-1β (pg/mL) | 253.2 ± 22.5; n=9 | 287.2 ± 25.9; n=10 | 498.2 ± 89.9; n=10 | 463.8 ± 95.4; n=10 | F(3,35)=3.13; P=0.0379 |
| IL-2 (ng/mL) | 5.2 ± 0.5; n=10 | 4.8 ± 0.2; n=10 | 5.1 ± 0.4; n=9 | 5.0 ± 0.3; n=10 | F(3,35)=0.20; P=0.8940 |
| IL-4 (pg/mL) | 346.1 ± 25.0; n=10 | 336.1 ± 14.8; n=10 | 446.3 ± 73.6; n=10 | 364.0 ± 24.7; n=10 | F(3,36)=1.46; P=0.2412 |
| IL-5 (ng/mL) | 1.0 ± 0.0; n=10 | 1.0 ± 0.0; n=10 | 1.0 ± 0.0; n=10 | 1.0 ± 0.0; n=10 | F(3,36)=0.23; P=0.8740 |
| IL-6 (ng/mL) | 0.9 ± 0.1; n=10 | 0.9 ± 0.1; n=10 | 1.2 ± 0.1; n=10 | 1.0 ± 0.1; n=10 | F(3,36)=1.76; P=0.1718 |
| IL-7 (ng/mL) | 3.0 ± 0.3; n=9 | 3.4 ± 0.3; n=10 | 5.8 ± 1.1; n=10 | 5.5 ± 1.3; n=10 | F(3,35)=2.52; P=0.0738 |
| IL-10 (ng/mL) | 2.2 ± 0.1; n=10 | 2.1 ± 0.1; n=10 | 2.4 ± 0.2; n=10 | 2.3 ± 0.1; n=10 | F(3,36)=0.78; P=0.5114 |
| IL-13 (pg/mL) | 432.4 ± 58.2; n=10 | 529.7 ± 107.7; n=10 | 830.8 ± 293.4; n=10 | 475.9 ± 72.7; n=10 | F(3,36)=1.22; P=0.3161 |
| IL-18 (ng/mL) | 16.4 ± 3.9; n=10 | 18.3 ± 3.4; n=10 | 16.6 ± 5.0; n=10 | 11.0 ± 1.6; n=10 | F(3,36)=0.73; P=0.5395 |
| G-CSF (pg/mL) | 17.5 ± 2.2; n=10 | 17.8 ± 1.9; n=10 | 24.3 ± 4.0; n=10 | 20.8 ± 2.3; n=10 | F(3,36)=1.33; P=0.2800 |
| GM-CSF (pg/mL) | 303.9 ± 30.7; n=9 | 366.9 ± 41.9; n=10 | 594.3 ± 116.1; n=10 | 536.6 ± 118.6; n=10 | F(3,35)=2.34; P=0.0899 |
| GRO/KC (pg/mL) | 544.3 ± 59.0; n=9 | 585.3 ± 64.8; n=10 | 712.0 ±111.3; n=10 | 665.9 ± 132.8; n=10 | F(3,35)=0.58; P=0.6308 |
| M-CSF (pg/mL) | 105.3 ± 9.6; n=10 | 99.5 ± 8.0; n=10 | 101.2 ± 9.9; n=10 | 117.4 ± 10.0; n=10 | F(3,36)=0.73; P=0.5412 |
| RANTES (ng/mL) | 1.9 ± 0.2; n=10 | 2.3 ± 0.3; n=10 | 2.5 ± 0.3; n=10 | 1.6 ± 0.2; n=10 | F(3,36)=3.22; P=0.0340 |
| VEGF (pg/mL) | 328.7 ± 41.3; n=9 | 369.4 ± 35.0; n=10 | 507.1 ± 95.7; n=10 | 388.8 ± 41.1; n=9 | F(3,34)=1.64; P=0.1977 |
| MCP-1 (ng/mL) | 1.4 ± 0.1; n=9 | 1.8 ± 0.2 n=10 | 2.2 ± 0.3 n=10 | 2.0 ± 0.4 n=10 | F(3,35)=1.41; P=0.2575 |
Supplementary Figure 9. In female rats from experiment 1, there was an overall effect of treatment on IL-1β and RANTES, while this was not attributed to any specific treatments. There were no other treatment effects on the remaining analyzed peptides. Data are presented as mean ±SEM

## Slide 12
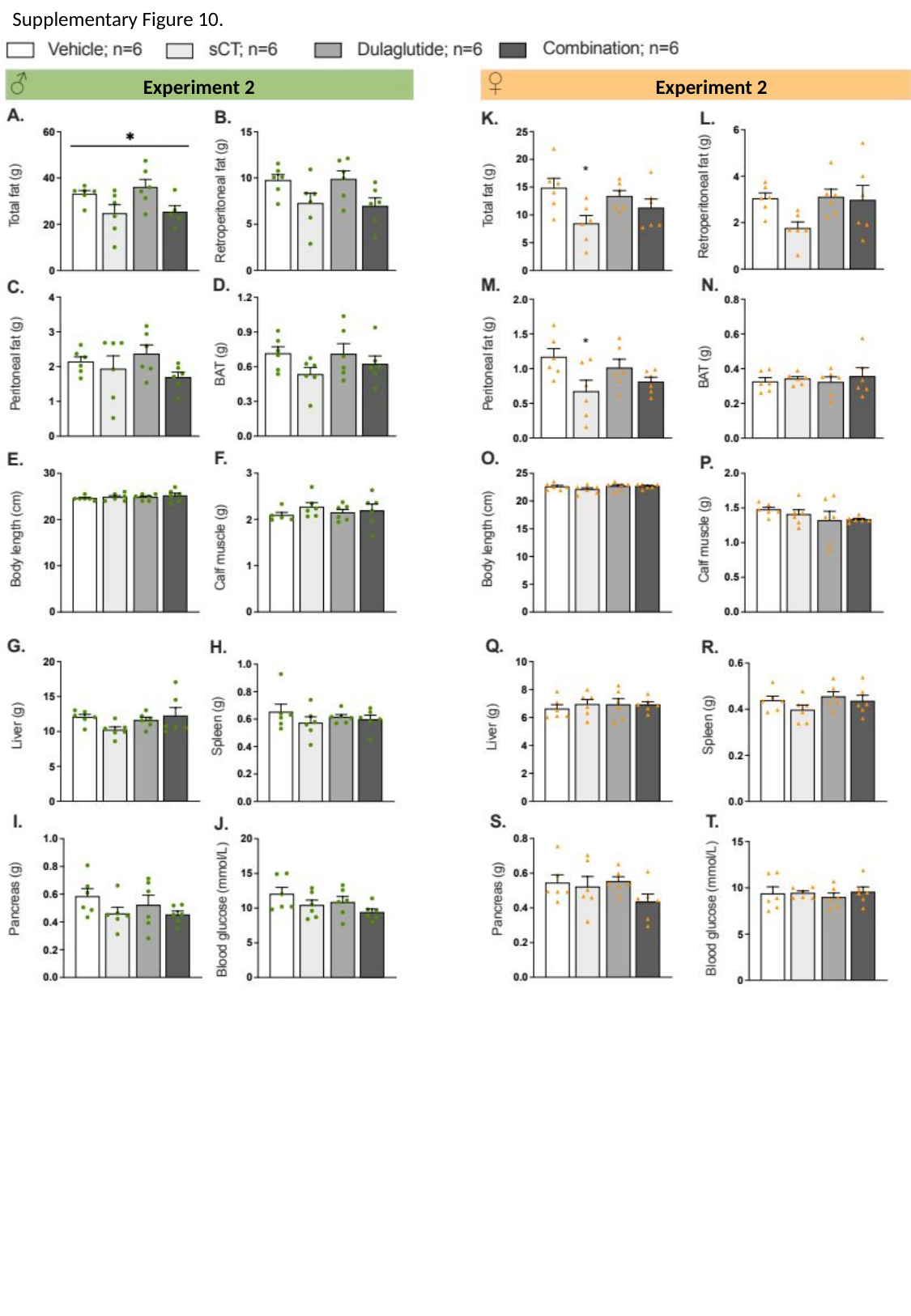

Supplementary Figure 10.
Experiment 2
Experiment 2

## Slide 13
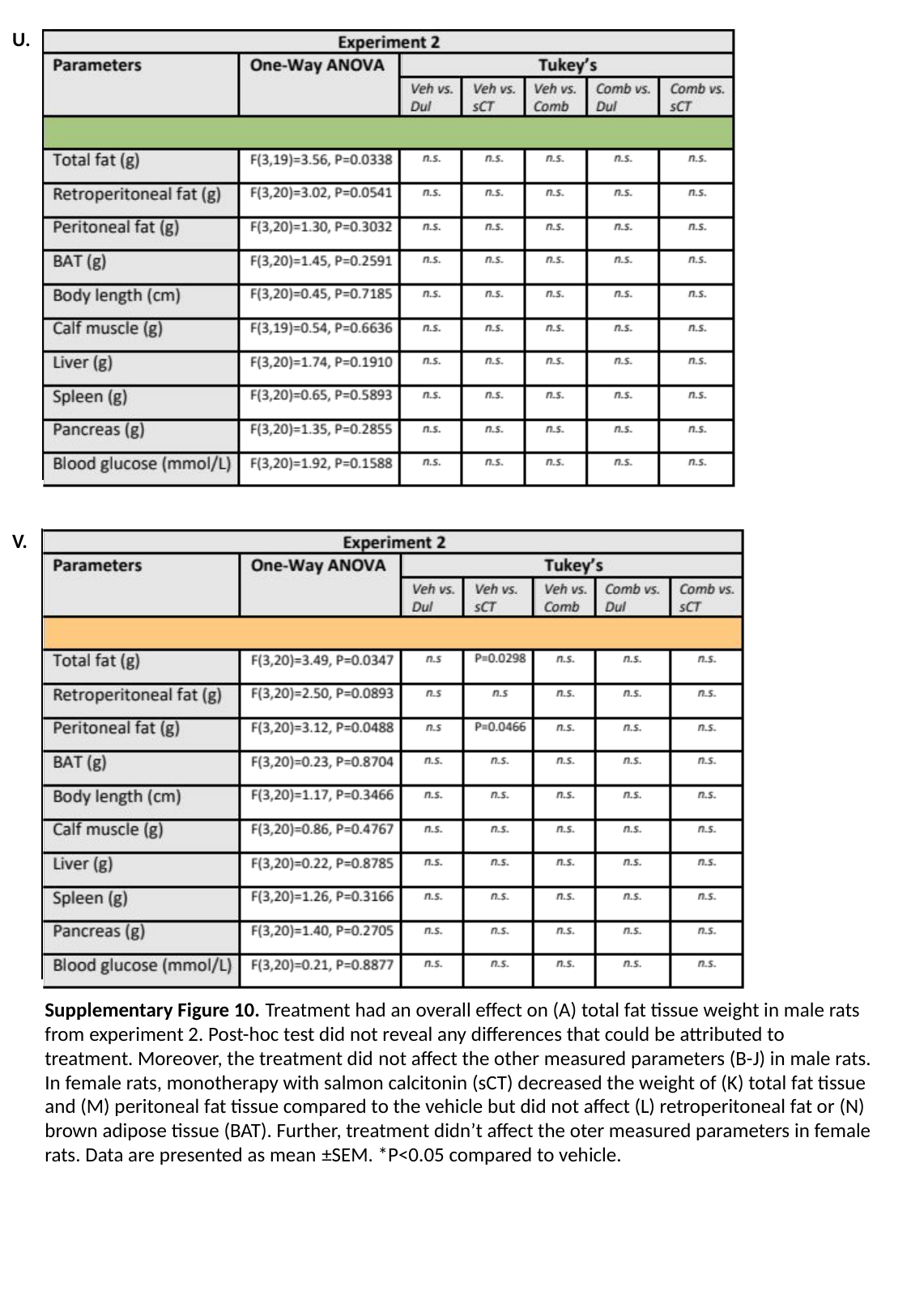

U.
V.
Supplementary Figure 10. Treatment had an overall effect on (A) total fat tissue weight in male rats from experiment 2. Post-hoc test did not reveal any differences that could be attributed to treatment. Moreover, the treatment did not affect the other measured parameters (B-J) in male rats. In female rats, monotherapy with salmon calcitonin (sCT) decreased the weight of (K) total fat tissue and (M) peritoneal fat tissue compared to the vehicle but did not affect (L) retroperitoneal fat or (N) brown adipose tissue (BAT). Further, treatment didn’t affect the oter measured parameters in female rats. Data are presented as mean ±SEM. *P<0.05 compared to vehicle.

## Slide 14
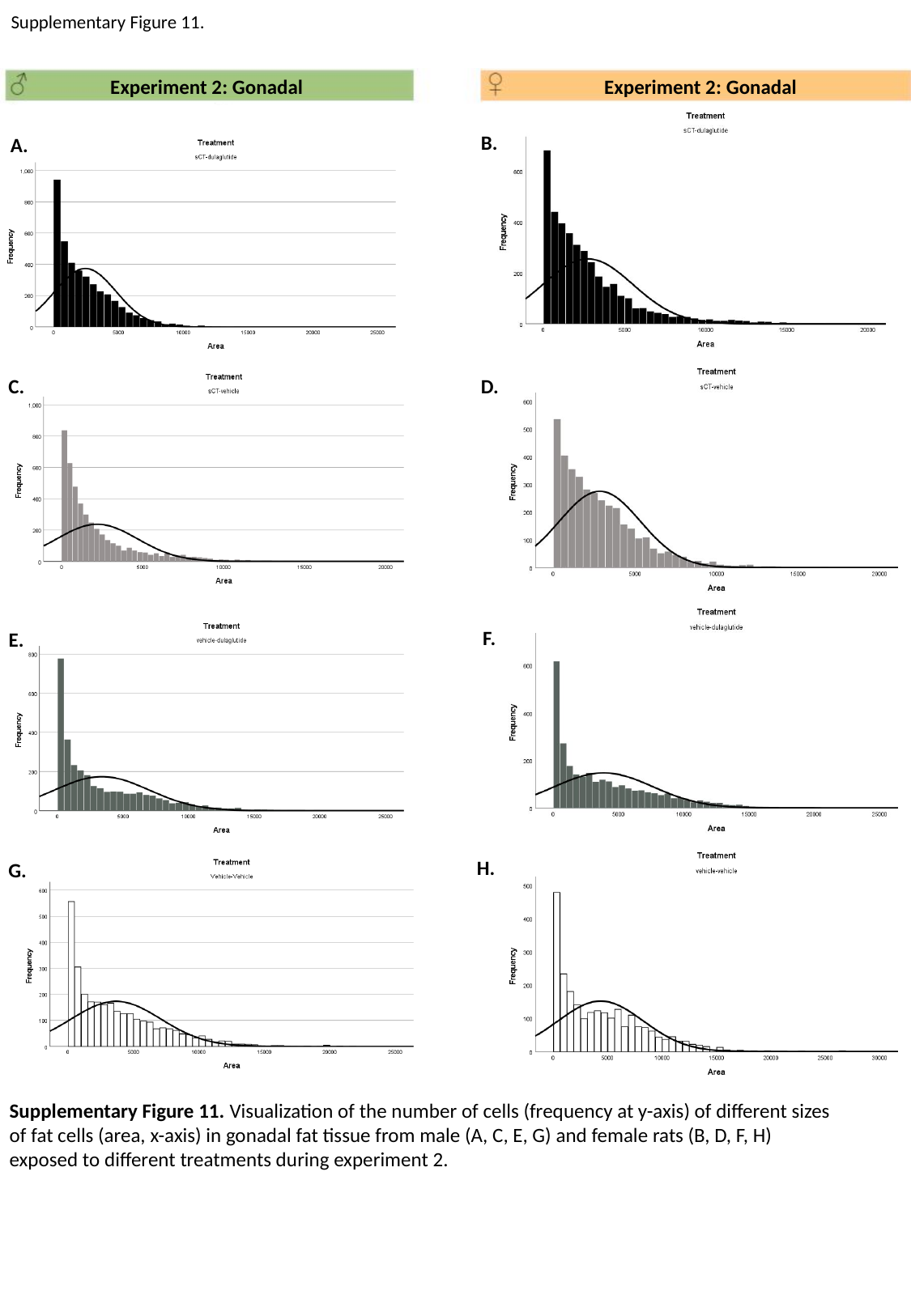

Supplementary Figure 11.
Experiment 2: Gonadal
Experiment 2: Gonadal
B.
A.
D.
C.
F.
E.
H.
G.
Supplementary Figure 11. Visualization of the number of cells (frequency at y-axis) of different sizes of fat cells (area, x-axis) in gonadal fat tissue from male (A, C, E, G) and female rats (B, D, F, H) exposed to different treatments during experiment 2.

## Slide 15
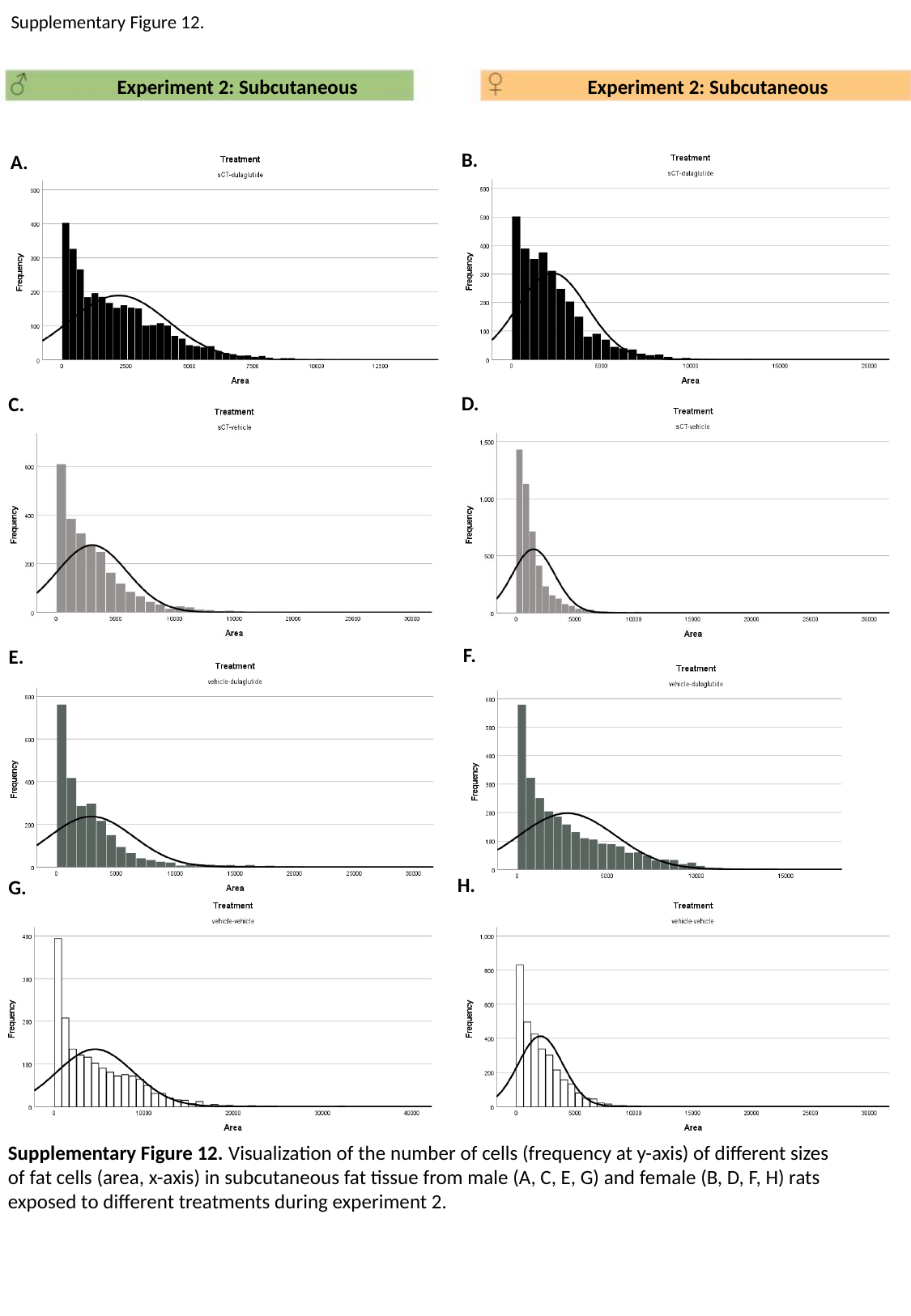

Supplementary Figure 12.
Experiment 2: Subcutaneous
Experiment 2: Subcutaneous
B.
A.
D.
C.
F.
E.
H.
G.
Supplementary Figure 12. Visualization of the number of cells (frequency at y-axis) of different sizes of fat cells (area, x-axis) in subcutaneous fat tissue from male (A, C, E, G) and female (B, D, F, H) rats exposed to different treatments during experiment 2.

## Slide 16
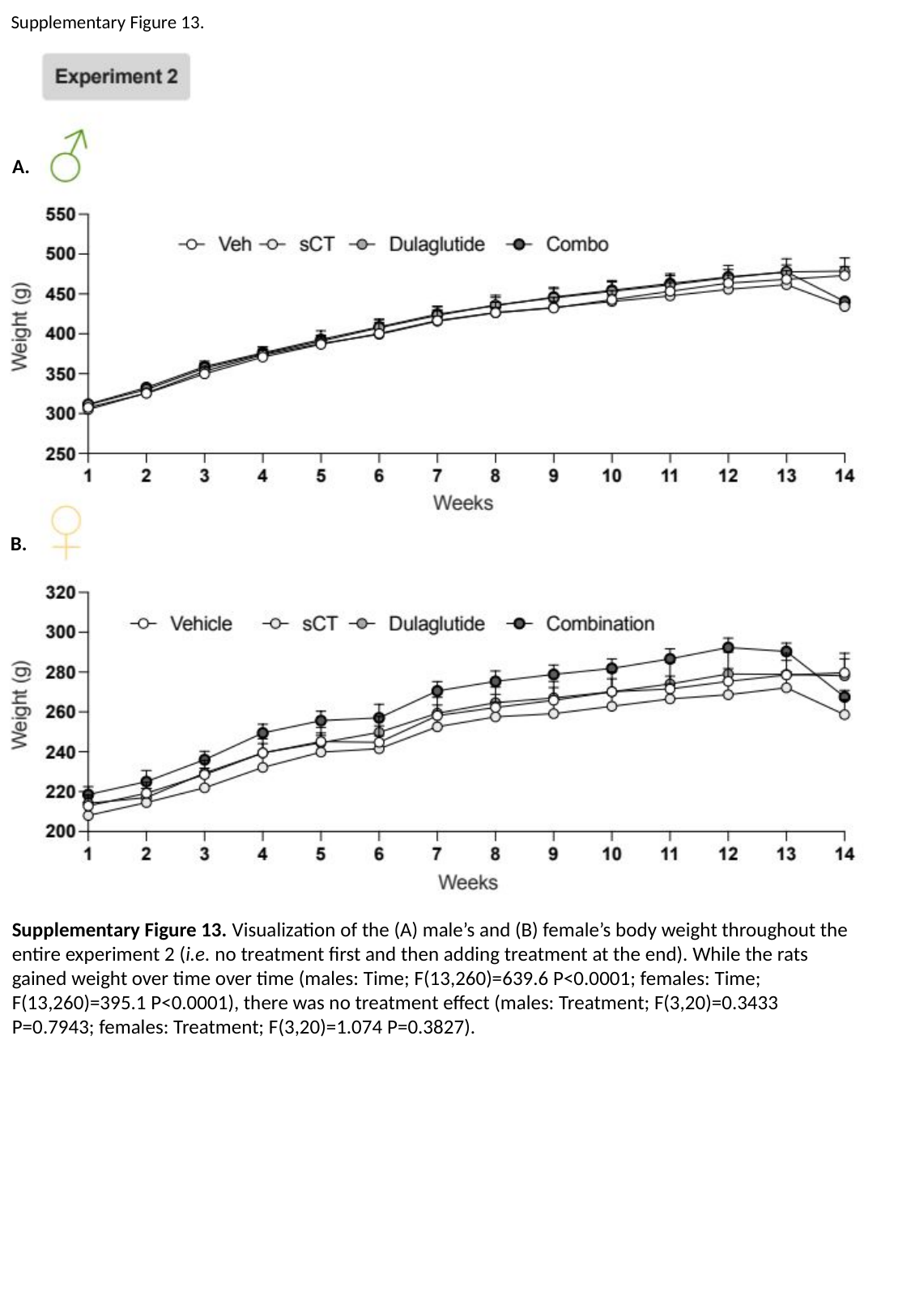

Supplementary Figure 13.
A.
B.
Supplementary Figure 13. Visualization of the (A) male’s and (B) female’s body weight throughout the entire experiment 2 (i.e. no treatment first and then adding treatment at the end). While the rats gained weight over time over time (males: Time; F(13,260)=639.6 P<0.0001; females: Time; F(13,260)=395.1 P<0.0001), there was no treatment effect (males: Treatment; F(3,20)=0.3433 P=0.7943; females: Treatment; F(3,20)=1.074 P=0.3827).
