# Supplementary material for: The combination of a glucagon-like peptide-1 and amylin receptor agonists reduces alcohol consumption in both male and female rats
Source: Acta Neuropsychiatr. 2024 Dec 6;37:e42. doi: 10.1017/neu.2024.58 (PMC13130276; doi:10.1017/neu.2024.58)
Supplement: Aranäs et al. supplementary material 2 — Aranäs et al. supplementary material [file S0924270824000589sup002.docx]

**Table 1. Effects on alcohol intake in male and female rats from experiments 1 and 2.**

| **Experiment 1** | | | | | | | | | | |
| --- | --- | --- | --- | --- | --- | --- | --- | --- | --- | --- |
|  |  | **Two-way ANOVA** | | **Tukey’s** | | | | | | |
|  |  |  |  | *Veh vs.*  *Dul* | *Veh vs. sCT* | | *Veh vs.*  *Comb* | | *Comb vs. Dul* | *Comb vs. sCT* |
| ♂  **M** | **Alcohol intake (g/kg)** | Time | F(5,180)=12.56, P<0.0001 | P<0.05, S4  P<0.01, S1 | P<0.05, S1 | | P<0.05, S4  P<0.01, S2  P<0.001, S1 | | n.s. | P<0.01, S4 |
|  |  | Treatment | F(3,36)=4.42 P=0.0096 |  |  |  |  |  |  |  |
|  |  | Interaction | F(15,180)=2.67, P=0.0011 |  |  |  |  |  |  |  |
| ♀ | **Alcohol intake (g/kg)** | Time | F(5,180)=21.57, P<0.0001 | P<0.05, S5  P<0.01, S1, S2 P<0.001, S4 | P<0.001, S1 | | P<0.05, S2, S4, S6  P<0.01, S5  P<0.001, S1 | | n.s. | n.s. |
|  |  | Treatment | F(3,36)=8.78 P=0.0002 |  |  |  |  |  |  |  |
|  |  | Interaction | F(15,180)=3.63, P<0.0001 |  |  |  |  |  |  |  |
| **Experiment 2** | | | | | | | | | | |
|  |  | **One-Way ANOVA** | | **Tukey’s** | | | | | | |
|  |  |  |  | *Veh vs.*  *Dul* | | *Veh vs. sCT* | | *Veh vs.*  *Comb* | *Comb vs. Dul* | *Comb vs. sCT* |
| ♂ | △ **Alcohol intake (g/kg)**  ***Alcohol drinking session 1*** | F(3,20)=0.78, P=0.5175 | | *n.s.* | | *n.s.* | | *n.s.* | *n.s.* | *n.s.* |
|  | △ **Alcohol intake (g/kg) *Alcohol drinking session 2*** | F(3,20)=3.32, P=0.0406 | | *n.s.* | | *n.s.* | | P=0.0315 | *n.s.* | *n.s.* |
| ♀ | △ **Alcohol intake (g/kg)**  ***Alcohol drinking session 1*** | F(3,20)=2.29, P=0.1095 | | *n.s.* | | *n.s.* | | *n.s.* | *n.s.* | *n.s.* |
|  | △ **Alcohol intake (g/kg)**  ***Alcohol drinking session 2*** | F(3,20)=3.38, P=0.0387 | | *n.s.* | | *n.s.* | | P=0.0263 | *n.s.* | *n.s.* |
|  |  | **Two-way ANOVA** | |  | | | | | | |
| ♂ | **Alcohol intake (g/kg)** | Time | F(4,80)=10.02, P<0.0001 | *n.s.* | *n.s.* | | *n.s.* | | *n.s.* | *n.s.* |
|  |  | Treatment | F(3,20)=0.16 P=0.9195 |  |  |  |  |  |  |  |
|  |  | Interaction | F(12,80)=1.19, P=0.3044 |  |  |  |  |  |  |  |
| ♀ | **Alcohol intake (g/kg)** | Time | F(4,80)=8,36, P<0.0001 | *n.s.* | *n.s.* | | *n.s.* | | *n.s.* | *n.s.* |
|  |  | Treatment | F(3,20)=2.00, P=0.1460 |  |  |  |  |  |  |  |
|  |  | Interaction | F(12,80)=0.83, P=0.6164 |  |  |  |  |  |  |  |

**Table 2. Effects on food intake in male and female rats from experiments 1 and 2.**

| **Experiment 1** | | | | | | | | |
| --- | --- | --- | --- | --- | --- | --- | --- | --- |
|  |  | **Two-way ANOVA** | | **Tukey’s** | | | | |
|  |  |  |  | *Veh vs.*  *Dul* | *Veh vs. sCT* | *Veh vs.*  *Comb* | *Comb vs. Dul* | *Comb vs. sCT* |
| ♂ | **Food intake (g)** | Time | F(5,180)=25.74, P<0.0001 | n.s. | P<0.01, S2  P<0.001, S1 | P<0.05, S4  P<0.001, S1, S2 | P<0.05, S6  P<0.001, S1, S2 | n.s. |
|  |  | Treatment | F(3,36)=23.03, P<0.0001 |  |  |  |  |  |
|  |  | Interaction | F(15,180)=7.016, P<0.0001 |  |  |  |  |  |
| ♀ | **Food intake (g)** | Time | F(5,180)=36.69, P<0.0001 | P<0.05, S1, S4 | P<0.05, S3, S4  P<0.001, S1 | P<0.01, S2, S6  P<0.001, S1, S3, S4 | P<0.01, S6  P<0.001, S1, S2, S3 | n.s. |
|  |  | Treatment | F(3,36)=36.05, P<0.0001 |  |  |  |  |  |
|  |  | Interaction | F(15,180)=7.18, P<0.0001 |  |  |  |  |  |
| **Experiment 2** | | | | | | | | |
|  |  | **Two-Way ANOVA** | | **Tukey’s** | | | | |
|  |  |  |  | *Veh vs.*  *Dul* | *Veh vs. sCT* | *Veh vs.*  *Comb* | *Comb vs. Dul* | *Comb vs. sCT* |
| ♂ | **Food intake (g)** | Time | F(6,120)=15.11, P<0.0001 | *n.s.* | P<0.001, S1, S2  P<0.01, S3, S4 | P<0.01, S5  P<0.001, S1, S2, S3 | P<0.0001, S1, S2, S3 S6 | P<0.05, S2 |
|  |  | Treatment | F(3,20)=31.95 P<0.0001 |  |  |  |  |  |
|  |  | Interaction | F(18,120)=6.90, P<0.0001 |  |  |  |  |  |
| ♀ | **Food intake (g)** | Time | F(6,120)=16.45, P<0.0001 |  | P<0.001, S2 | P<0.001, S1, S2 | P<0.01, S1  P<0.001, S2 |  |
|  |  | Treatment | F(3,20)=18.32 P<0.0001 |  |  |  |  |  |
|  |  | Interaction | F(18,120)=6.34, P<0.0001 |  |  |  |  |  |

**Table 3**. **Effects on body weight in male and female rats from experiments 1 and 2.**

| **Experiment 1** | | | | | | | | |
| --- | --- | --- | --- | --- | --- | --- | --- | --- |
|  |  | **Two-way ANOVA** | | **Tukey’s** | | | | |
|  |  |  |  | *Veh vs.*  *Dul* | *Veh vs. sCT* | *Veh vs.*  *Comb* | *Comb vs. Dul* | *Comb vs. sCT* |
| ♂ | **Body weight change (g)** | Time | F(4,144)=33.91, P<0.0001 | *P<0.01, S4*  *P<0.0001, S1* |  | *P<0.0001, S1* | *P<0.0001, S1* | *P<0.05, S1* |
|  |  | Treatment | F(3,36)=33.10, P<0.0001 |  |  |  |  |  |
|  |  | Interaction | F(12,144)=8.53, P<0.0001 |  |  |  |  |  |
| ♀ | **Body weight change (g)** | Time | F(4,144)=88.01, P<0.0001 | *P<0.05, S1*  *P<0.01, S4* | *P<0.0001, S1* | *P<0.0001, S1, S4* | *P<0.0001, S1* | *P<0.05, S4*  *P<0.0001, S1* |
|  |  | Treatment | F(3,36)=26.83, P<0.0001 |  |  |  |  |  |
|  |  | Interaction | F(12,144)=17.11, P<0.0001 |  |  |  |  |  |
| **Experiment 2** | | | | | | | | |
|  |  | **Two-Way ANOVA** | | **Tukey’s** | | | | |
|  |  |  |  | *Veh vs.*  *Dul* | *Veh vs. sCT* | *Veh vs.*  *Comb* | *Comb vs. Dul* | *Comb vs. sCT* |
| ♂ | **Body weight change (g)** | Time | F(6,120)=36.96, P<0.0001 | *P<0.0001, S1* | *P<0.01, S1* | *P<0.0001, S1* | *P<0.05, S2*  *P<0.0001, S1* | *P<0.0001, S1* |
|  |  | Treatment | F(3,20)=16.41 P<0.0001 |  |  |  |  |  |
|  |  | Interaction | F(18,120)=9.67, P<0.0001 |  |  |  |  |  |
| ♀ | **Body weight change (g)** | Time | F(6,120)=14.69, P<0.0001 |  | *P<0.01, S1* | *P<0.0001, S1, S2* | *P<0.05, S1*  *P<0.0001, S2* |  |
|  |  | Treatment | F(3,20)=8.49 P=0.0008 |  |  |  |  |  |
|  |  | Interaction | F(18,120)=4.12, P<0.0001 |  |  |  |  |  |

**Table 4. Effects on gonadal and subcutaneous fat tissues from male and female rats of experiment 2.**

|  | **Morphology** | | | | | | |
| --- | --- | --- | --- | --- | --- | --- | --- |
|  | **Parameters** | **One-Way ANOVA** | **Tukey’s** | | | | |
|  |  |  | *Veh vs.*  *Dul* | *Veh vs. sCT* | *Veh vs.*  *Comb* | *Comb vs. Dul* | *Comb vs. sCT* |
| ♂  **M** | **Weight (g)** *Gonadal* | F (3, 20) = 3.15  P=0.0476* | n.s. | n.s. | n.s. | n.s. | n.s. |
|  | **Number of cells** *Gonadal* | F (3, 32) = 3.10  P=0.0405* | n.s. | n.s. | n.s. | n.s. | n.s. |
|  | **Area of cells** *Gonadal* | F (3, 32) = 3.94  P=0.0168* | n.s. | n.s. | P=0.0297 | n.s. | n.s. |
|  | **Contrast** *Gonadal* | F (3, 32) = 1.59  P=0.2106 | n.s. | n.s. | n.s. | n.s. | n.s. |
|  | **Weight (g)** *Subcutaneous* | F (3, 19) = 2.54 P=0.0873 | n.s. | n.s. | n.s. | n.s. | n.s. |
|  | **Number of cells** *Subcutaneous* | F (3, 32) = 5.10  P=0.0054** | n.s. | n.s. | P=0.0023 | n.s. | n.s. |
|  | **Area of cells** *Subcutaneous* | F (3, 32) = 3.22 P=0.0354* | n.s. | n.s. | P=0.0250 | n.s. | n.s. |
|  | **Contrast** *Subcutaneous* | F (3, 32) = 4.01  P=0.0157* | P=0.0104 | n.s. | n.s. | n.s. | n.s. |
| ♀ | **Weight (g)** *Gonadal* | F (3, 20) = 2.93  P=0.0587 | n.s. | n.s. | n.s. | n.s. | n.s. |
|  | **Number of cells** *Gonadal* | F (3, 32) = 4.62  P=0.0085** | n.s. | P=0.0363 | P=0.0195 | n.s. | n.s. |
|  | **Area of cells** *Gonadal* | F (3, 32) = 3.08  P=0.0414* | n.s. | n.s. | n.s. | n.s. | n.s. |
|  | **Contrast** *Gonadal* | F (3, 32) = 2.07  P=0.1232 | n.s. | n.s. | n.s. | n.s. | n.s. |
|  | **Weight (g)** *Subcutaneous* | F (3, 20) = 3.50 P=0.0346* | n.s. | n.s. | n.s. | n.s. | n.s. |
|  | **Number of cells** *Subcutaneous* | F (3, 32) = 3.87  P=0.0182* | n.s. | n.s. | n.s. | n.s. | n.s. |
|  | **Area of cells** *Subcutaneous* | F (3, 32) = 2.73 P=0.0600 | n.s. | n.s. | n.s. | n.s. | n.s. |
|  | **Contrast** *Subcutaneous* | F (3, 32) = 1.13  P=0.3530 | n.s. | n.s. | n.s. | n.s. | n.s. |
